# HADHA-mediated regulation of JAK/STAT3 signaling in glioblastoma: a metabolic-epigenetic axis

**DOI:** 10.1038/s41420-025-02660-0

**Published:** 2025-08-01

**Authors:** Kan Wang, Yifei Xiao, Jinxin Wan, Yuanqi Chu, Ruipeng Zheng, Fengjun Lv, Guang Yang, Mingchun Yang, Haitao Ge, Yuwen Song, Yu Cheng

**Affiliations:** 1 Department of Neurosurgery, The First Affiliated Hospital of Harbin Medical University, Harbin City, Heilongjiang Province China; 2 Department of neurosurgery, The Fourth Affiliated Hospital of Harbin Medical University, Harbin City, Heilongjiang Province China; 3 Department of Neurosurgery, Guangdong Provincial People’s Hospital, Zhuhai Hospital (Jinwan Central Hospital of Zhuhai), Zhuhai City, Guangdong Province China; 4 Department of Pathology, XD Group hospital, Xi’an, China; 5 Department of Neurosurgery, The Second Affiliated Hospital of Fujian Medical University, Quanzhou, Fujian Province China

**Keywords:** CNS cancer, Prognostic markers, Oncogenes, Tumour biomarkers

## Abstract

Glioblastoma multiforme (GBM) is one of the most aggressive forms of brain cancer, characterized by rapid growth and resistance to conventional therapies. This study investigates the role of HADHA, a key enzyme in fatty acid β-oxidation, in the progression of GBM. we show that the overexpression of HADHA in GBM correlates with a poor prognosis in patients and plays a role in promoting tumor growth and invasion. Mechanistically, HADHA regulates the JAK/STAT3 signaling pathway through modulation of H3K27ac histone acetylation. Knockdown of HADHA results in decreased acetyl-CoA levels, leading to reduced H3K27ac modification and subsequent inhibition of JAK/STAT3 activation. Furthermore, we show that the small molecule JIB-04, which targets HADHA, inhibits GBM cell proliferation and invasion both in vitro and in vivo. Our findings highlight the importance of targeting metabolic enzymes in cancer therapy and suggest that HADHA could represent a potential new therapeutic target for GBM. By targeting the metabolic-epigenetic pathway, this strategy presents a promising approach for treating this devastating disorder.

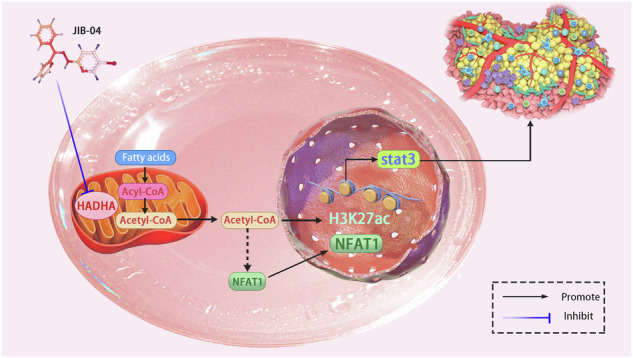

## Introduction

GBM is one of the most aggressive and malignant tumors of the central nervous system, characterized by its rapid growth, high invasiveness, and resistance to conventional therapies [1]. Although there have been notable advancements in treatment options, such as surgery, radiation, and chemotherapy, the prognosis for GBM patients continues to be poor because of the tumor’s intrinsic heterogeneity and resistance to therapies [2]. The aggressive nature of GBM is influenced by complex signaling networks and metabolic pathways that regulate tumor growth and survival, highlighting the need for a deeper understanding of these mechanisms to develop more effective therapeutic strategies.

Recent research has revealed a critical link between metabolism and epigenetic regulation in cancer, including GBM [3]. One of the key metabolic pathways that influence epigenetic modifications is the degradation of fatty acids, particularly through the enzyme HADHA (long-chain acyl-CoA dehydrogenase). HADHA is involved in the β-oxidation of fatty acids and is a crucial regulator of intracellular acetyl-CoA levels, which are directly implicated in the process of histone acetylation [4]. Acetyl-CoA, as a substrate for histone acetyltransferases, promotes the acetylation of histones, such as H3K27ac, a modification associated with gene activation and tumorigenesis [5]. Altered acetyl-CoA metabolism has been recognized as a driving force behind the aberrant gene expression observed in GBM.

The H3K27ac modification, a key marker of active gene promoters, plays an essential role in controlling the expression of genes that contribute to tumor growth and invasion [6]. It has been shown that H3K27ac modification is particularly important in cancer cells, where it can activate oncogenes and promote the malignant features of tumors [7]. In GBM, changes in histone acetylation, driven by enzymes like HADHA, can facilitate the activation of signaling pathways that are critical for tumor progression.

One such pathway is the JAK/STAT3 signaling pathway, a pivotal cascade involved in regulating cell survival, proliferation, and immune evasion [8]. STAT3, the main effector of JAK signaling, is frequently hyperactivated in GBM, leading to enhanced tumor cell proliferation and resistance to apoptosis [9]. Recent studies have suggested that metabolic reprogramming, particularly the regulation of acetyl-CoA and histone acetylation, may directly influence the activation of JAK/STAT3 signaling in GBM.

This study aims to explore how HADHA, through its regulation of H3K27ac modification, activates the JAK/STAT3 pathway in GBM. By investigating the role of HADHA in modifying the epigenetic landscape of GBM and its potential to regulate key oncogenic pathways, this research seeks to uncover novel therapeutic strategies that target the intersection of metabolism, epigenetics, and signaling pathways in GBM. Understanding the molecular mechanisms by which HADHA modulates H3K27ac and activates JAK/STAT3 could provide valuable insights into developing effective treatments for this devastating disease.

## Result

### HADHA is overexpressed in GBM and associated with poor prognosis

**Fig. 1:**
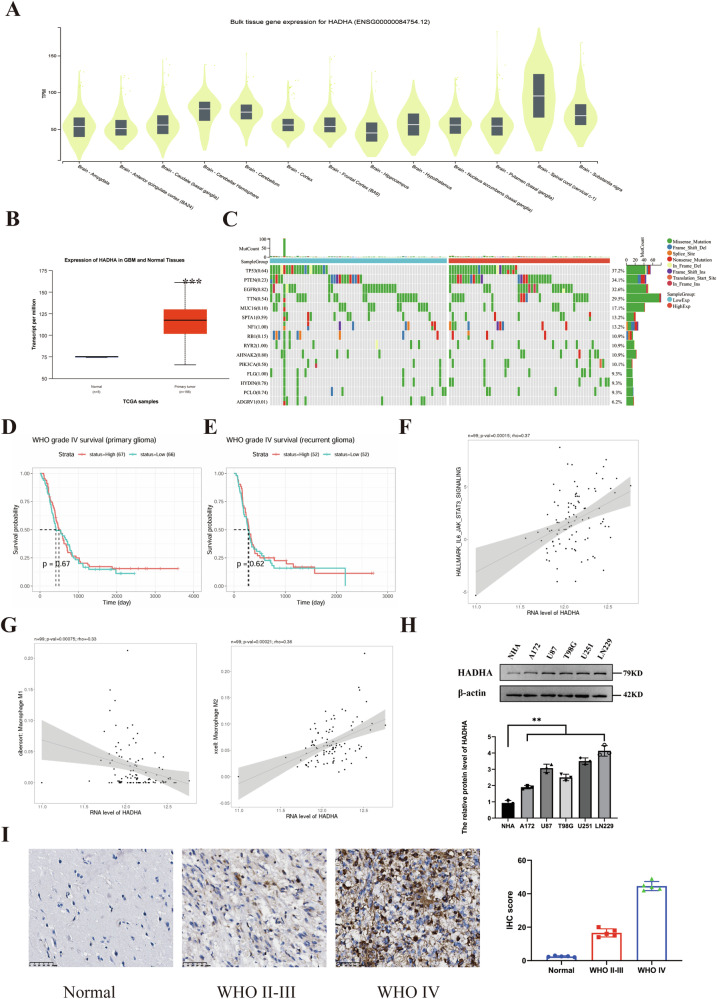
Expression and clinical correlation of HADHA in GBM. **A** HADHA expression in various brain regions based on GTEx data, showing higher levels in cerebellum and spinal cord (*P* < 0.05). **B** HADHA is significantly overexpressed in GBM compared to normal brain tissues (*P* < 0.001, TCGA data). **C** Higher mutation rates of TP53 and PTEN in GBM with high HADHA expression (*P* < 0.05). **D**, **E** Kaplan-Meier survival analysis of GBM patients with high and low HADHA mRNA levels in the CGGA database. No significant difference in survival is observed between primary and recurrent GBM patients with high HADHA expression compared to those with low expression. **F** Positive correlation between HADHA mRNA and JAK/STAT3 pathway activation (*P* < 0.05). **G** Negative correlation with M1 macrophages (*P* < 0.05) and positive correlation with M2 macrophages (*P* < 0.01). **H** High HADHA protein expression in glioma cell lines (*P* < 0.001). **I** Higher HADHA protein levels in GBM tissues compared to LGG and normal brain, localized in the cytoplasm (*P* < 0.001).

We analyzed the expression levels of HADHA in normal human brain tissues using the GTEx database, finding relatively higher expression in the cerebellum and spinal cord (cervical c-1), while expression in the hippocampus was relatively lower (Fig. 1A). Based on the The Cancer Genome Atlas (TCGA) database, HADHA expression in GBM was significantly higher than in normal tissues (Fig. 1B). By comparing mutation frequencies at different HADHA expression levels, we found that the mutation rates of TP53 and PTEN genes were higher in the high HADHA expression group than in the low expression group, revealing the potential role of this gene in GBM and its interactions with other genes (Fig. 1C). Kaplan-Meier analysis of patient survival rates in the Chinese Glioma Genome Atlas (CGGA) database showed no significant difference in survival between primary and recurrent GBM patients with high vs. low HADHA mRNA levels (Fig. 1D, E). Furthermore, correlation analysis of HADHA mRNA with phenotypes using LinkedOmicsKB showed a positive correlation with the JAK/STAT3 pathway (Fig. 1F), a negative correlation with M1 macrophages, and a positive correlation with M2 macrophages (Fig. 1G). We also validated HADHA protein expression levels in normal brain astrocytes and glioma cells, finding that HADHA was generally highly expressed in glioma cell lines (Fig. 1H). To verify elevated HADHA protein levels in GBM, we conducted IHC staining for HADHA in an independent cohort, including 10 glioma and 5 normal brain tissue samples. Results showed higher HADHA protein levels in GBM compared to LGG and normal brain tissues, with HADHA localized to the cytoplasm (Fig. 1I), matching our previous findings in glioma cells.

### HADHA knockdown inhibits GBM growth both in vitro and in vivo

**Fig. 2:**
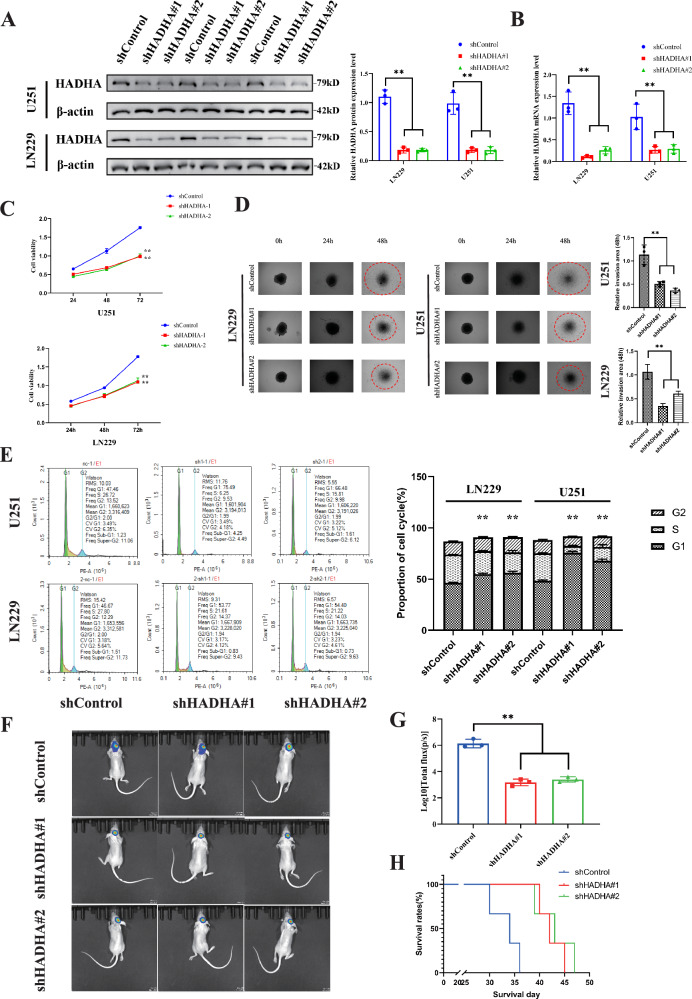
HADHA knockdown inhibits GBM growth both in vitro and in vivo. **A** Western blot analysis confirming the protein expression levels of HADHA in U251 and LN229 cells. HADHA protein levels were significantly decreased in shHADHA#1 and shHADHA#2 compared to shControl (*P* < 0.01). **B** qPCR analysis showing the knockdown efficiency of HADHA in U251 and LN229 cells. The expression levels of HADHA mRNA were significantly reduced in shHADHA#1 and shHADHA#2 compared to shControl (*P* < 0.001). **C** Cell viability assay results showing decreased cell viability in HADHA-knockdown U251 and LN229 cells compared to shControl at 24 and 48 h (*P* < 0.05). **D** Cancer Cell Spheroid Invasion Assay demonstrating reduced invasion capability of HADHA-knockdown U251 and LN229 cells compared to shControl (*P* < 0.01). **E** Flow cytometry analysis of cell cycle distribution showing an increased proportion of cells in the G1 phase in HADHA-knockdown U251 and LN229 cells compared to shControl (*P* < 0.05). **F** Bioluminescent imaging showing tumor growth in shControl and shHADHA groups of mice. **G** In vivo tumor growth analysis in a mouse orthotopic model. Tumor volume was significantly smaller in the shHADHA group compared to the shControl group at day 30 (*P* < 0.001). **H** Kaplan-Meier survival curve showing increased overall survival in mice with shHADHA tumors compared to shControl tumors (*P* < 0.05).

To investigate the role of HADHA in glioma, we knocked down HADHA in the high-expressing cell lines LN229 and U251, generating stable shRNA-expressing cell populations (shControl, shHADHA#1, and shHADHA#2). qPCR and Western blot analyses confirmed successful HADHA knockdown, with significantly reduced mRNA and protein expression levels in LN229 and U251 cells compared to controls (Fig. 2A, B). Cell viability was significantly decreased in HADHA-shRNA LN229 and U251 cell lines compared to controls (Fig. 2C). The 3D invasion capability of HADHA-knockdown LN229 and U251 cells was significantly reduced in the Cancer Cell Spheroid Invasion Assay (Fig. 2D). Flow cytometry analysis of the cell cycle revealed a significant increase in the proportion of cells in the G1 phase in HADHA-shRNA LN229 and U251 cell lines (Fig. 2E), suggesting that HADHA depletion may reduce cell proliferation by inducing G1 cell cycle arrest. Intracranial injection of shHADHA or shControl LN229 cells into mice established an orthotopic tumor model to explore the impact of HADHA on tumor growth in vivo (Fig. 2F). After 30 days, in vivo imaging showed that tumor growth was significantly weaker in the shHADHA group than in the control group (Fig. 2G), and the overall survival of mice in the shHADHA group was increased (Fig. 2H).

### HADHA mediates histone acetylation modifications to regulate the JAK/STAT pathway

**Fig. 3:**
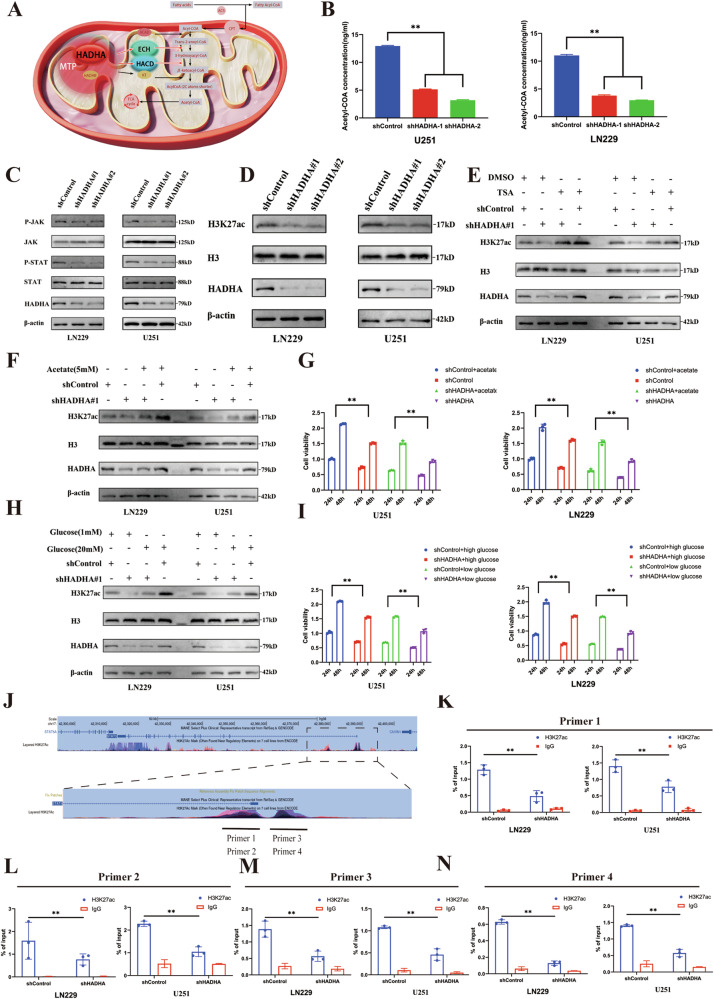
Mechanism of HADHA-mediated regulation of JAK/STAT3 signaling through H3K27ac modification. **A** Schematic Representation of Fatty Acid β-Oxidation and Its Integration with the Mitochondrial Trifunctional Protein. **B** Bar graphs representing the acetyl-CoA concentrations in U251 and LN229 glioblastoma cells following HADHA knockdown. The concentrations are significantly reduced in HADHA-knockdown cells compared to the shControl group. **C** Western blot analysis of JAK/STAT3 pathway components in U251 and LN229 cells. HADHA knockdown led to decreased P-JAK and P-STAT3, while JAK and STAT3 protein levels remained unchanged (*P* < 0.05). **D** Knockdown of HADHA reduced H3K27ac levels in LN229 and U251 cells. **E** Rescue experiment with TSA showing partial restoration of H3K27ac levels in HADHA-knockdown cells (*P* < 0.05). **F** Western blot analysis demonstrates that the supplementation of acetic acid restores the levels of H3K27ac in HADHA-knockdown cells. **F** Western blot analysis demonstrates that the supplementation of acetic acid restores the levels of H3K27ac in HADHA-knockdown cells. **G** The CCK-8 assay indicates that treatment with acetic acid restores the cellular viability of HADHA-knockdown U251 and LN229 cells. **H** Western blot analysis shows that the supplementation of glucose restores the levels of H3K27ac in HADHA-knockdown cells. **I** The CCK-8 analysis indicates that treatment with glucose restores the cellular vitality of HADHA-knockdown U251 and LN229 cells. **J** Schematic diagram of the HADHA gene and the location of primers used for ChIP-qPCR analysis. Primer 1, 2, 3, and 4 are designed to amplify specific regions of the HADHA promoter and enhancer. **K**–**N** ChIP-qPCR results showing the enrichment of H3K27ac at the HADHA promoter and enhancer regions in U251 and LN229 cells. HADHA knockdown significantly reduced H3K27ac enrichment at these regions (*P* < 0.01).

HADHA is the α subunit of the mitochondrial trifunctional protein, which plays a crucial role in the β-oxidation of fatty acids. It affects the activities of enoyl-CoA hydratase (ECH) and L-β-hydroxyacyl-CoA dehydrogenase, participating in steps 2 and 3 of fatty acid oxidation (Fig. 3A). As a key enzyme in the fatty acid oxidation pathway, HADHA converts fatty acyl-CoA into acetyl-CoA. Acetyl-CoA, one of the main products of fatty acid oxidation, not only serves as an intermediate metabolite in biosynthesis and catabolic processes but also plays a significant role in promoting epigenetic modifications, which are critical for GBM progression. Acetyl-CoA, as a donor of acetyl groups, directly influences histone acetylation, which in turn affects chromatin structure and downstream gene transcription. Localized histone acetylation creates a more open chromatin structure, facilitating DNA access for transcription machinery and promoting gene expression.

We found that knocking down HADHA led to a decrease in intracellular acetyl-CoA levels (Fig. 3B). Previous experiments have shown that HADHA is closely associated with the JAK/STAT signaling pathway. Therefore, after knocking down HADHA, we assessed the protein levels of JAK/STAT in cells and found a significant reduction in P-JAK and P-STAT, while JAK and STAT remained unchanged. This suggests that HADHA may exert its effect through the JAK/STAT pathway (Fig. 3C).

To identify the molecular mechanism through which HADHA induces STAT expression, we examined H3K27ac marks in the STAT promoter region using the UCSC Genome Browser and found that H3K27ac modifications might regulate STAT transcription. Knockdown of HADHA reduced H3K27ac levels in LN229 and U251 cells (Fig. 3D), and treatment with the histone deacetylase inhibitor TSA rescued this effect (Fig. 3E).

To explore the role of HADHA in acetyl-CoA-induced H3K27ac, we varied the glucose and acetate concentrations in the culture medium and measured H3K27ac levels. Glucose and acetate are important precursors of acetyl-CoA. Glucose is metabolized through glycolysis and the tricarboxylic acid cycle to generate acetyl-CoA, while acetate can be directly converted into acetyl-CoA. By altering the concentrations of these two metabolites, we can regulate the intracellular levels of acetyl-CoA, which in turn affects histone acetylation and allows us to investigate their roles in HADHA-regulated epigenetic modifications. The results indicated that increasing the concentrations of glucose and acetate effectively restored the reduced H3K27ac levels (Fig. 3F, H). Supplementing glucose and acetate rescued the growth inhibition caused by HADHA knockdown (Fig. 3G, I). In conclusion, HADHA-mediated elevation of acetyl-CoA promotes an increase in H3K27ac levels.

We performed ChIP analysis on lysates from HADHA knockdown cells using primers targeting the H3K27ac region of the STAT promoter (Fig. 3J). The results showed lower levels of STAT fragments captured by the H3K27ac antibody-agarose complex in HADHA knockdown cells compared to the control (Fig. 3K–N). These results suggest that silencing HADHA weakens H3K27ac binding to the STAT promoter, indicating that HADHA-mediated changes in H3K27ac influence STAT transcription.

**Fig. 4:**
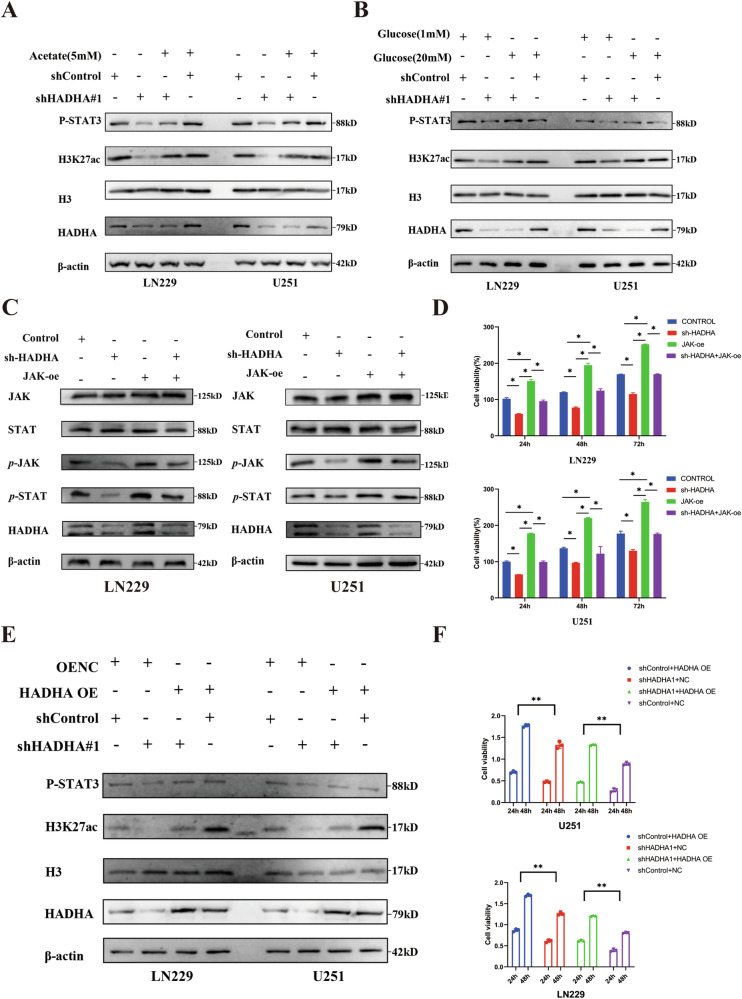
Impact of rescue experiments on HADHA-knockdown U251 and LN229 Cells. **A** Western blot analysis demonstrates the changes in levels of P-STAT3 and H3K27ac in HADHA-knockdown U251 and LN229 cells following the supplementation of acetate. Compared to the shControl, the addition of acetate partially restores the levels of P-STAT3 and H3K27ac (*P* < 0.05). **B** Western blot analysis shows the changes in the levels of P-STAT3 and H3K27ac in HADHA-knockdown U251 and LN229 cells after the supplementation of glucose. Compared to the shControl, the addition of glucose partially restores the levels of P-STAT3 and H3K27ac (*P* < 0.05). **C** Western Blot Analysis of JAK/STAT3 Signaling Pathway in LN229 and U251 Cells After HADHA Knockdown and JAK Overexpression. **D** CCK-8 analysis of JAK/STAT3 Signaling Pathway in LN229 and U251 Cells After HADHA Knockdown and JAK Overexpression. **E** The impact of HADHA re-expression on the levels of P-STAT3 and H3K27ac proteins after HADHA knockdown. **F** The effect of HADHA re-expression on the viability of U251 and LN229 cells after HADHA knockdown.

We adjusted glucose and acetate concentrations in the culture medium to determine if these metabolites could rescue H3K27ac levels and STAT expression. Treatment with acetate (Fig. 4A) or higher concentrations of glucose (Fig. 4B) restored STAT protein levels.

We conducted rescue experiments by overexpressing JAK after HADHA knockdown (Fig. 4C). Overexpression of JAK rescued tumor growth in the shHADHA group of LN229 and U251 cells (Fig. 4D). These results further confirmed the role of the HADHA/JAK/STAT axis in glioma growth.

To further confirm that these results depend on functional HADHA, we re-expressed HADHA after knocking it down. Western blotting showed the rescuing effects on H3K27ac modification and STAT protein levels (Fig. 4E), as well as a recovery of cell proliferation (Fig. 4F).

### The nuclear translocation of NFAT1 promotes histone acetylation modifications

**Fig. 5:**
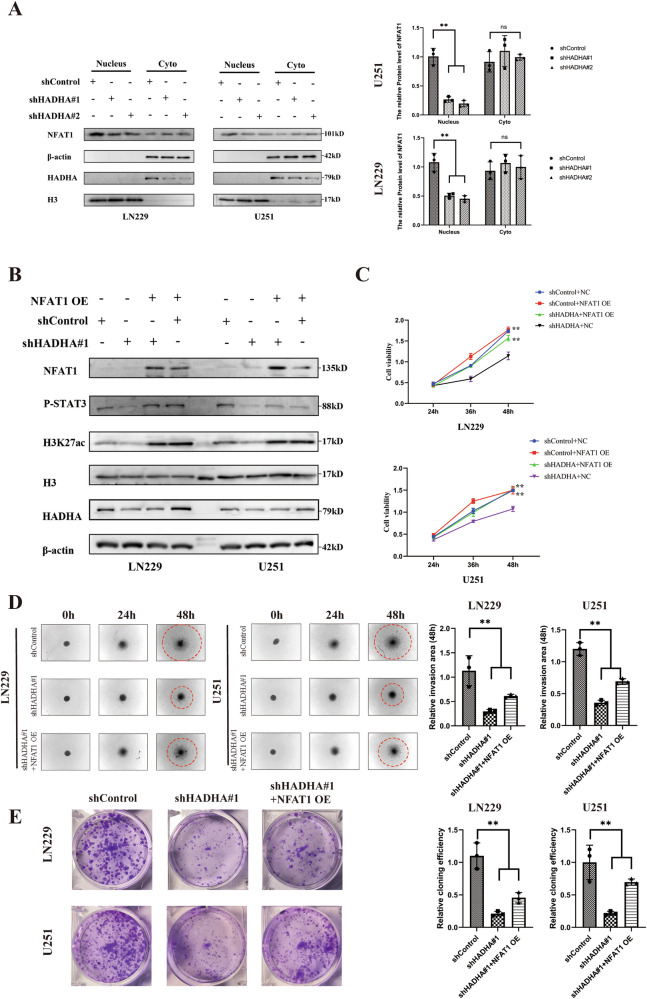
The nuclear translocation of NFAT1 promotes histone acetylation modifications. **A** The changes in NFAT1 localization in the cytoplasm and nucleus of LN229 and U251 cells after HADHA knockdown. **B** The impact of NFAT1 overexpression on the levels of P-STAT3 and H3K27ac in LN229 and U251 cells after HADHA knockdown. **C** The effect of NFAT1 overexpression on the viability changes of LN229 and U251 cells after HADHA knockdown. **D** The Cancer Cell Spheroid Invasion analysis of the impact of NFAT1 overexpression on the invasive capacity of LN229 and U251 cells after HADHA knockdown. **E** The effect of NFAT1 overexpression on colony formation in LN229 and U251 cells after HADHA knockdown.

NFAT1 regulates site-specific histone acetylation, including H3K27ac. In LN229 and U251 cells, HADHA knockdown inhibited NFAT1 nuclear accumulation (Fig. 5A). Since glucose and acetate are known substrates for acetyl-CoA production, supplementation with these metabolites restored NFAT1 levels in the nucleus without affecting total NFAT1 expression, supporting our findings (Figure S1A-C). Additionally, overexpressing NFAT1 reversed the decrease in H3K27ac and P-STAT protein levels caused by HADHA knockdown (Fig. 5B).

CCK-8 assays revealed that NFAT1 overexpression could rescue the proliferative ability of cells in the shHADHA group (Fig. 5C). Moreover, following NFAT1 overexpression, the 3D invasion ability and colony formation ability of the shHADHA group cells were also restored (Fig. 5D, E).

### JIB-04 disrupts the HADHA/JAK/STAT axis and holds potential as a therapeutic agent for treating GBM

**Fig. 6:**
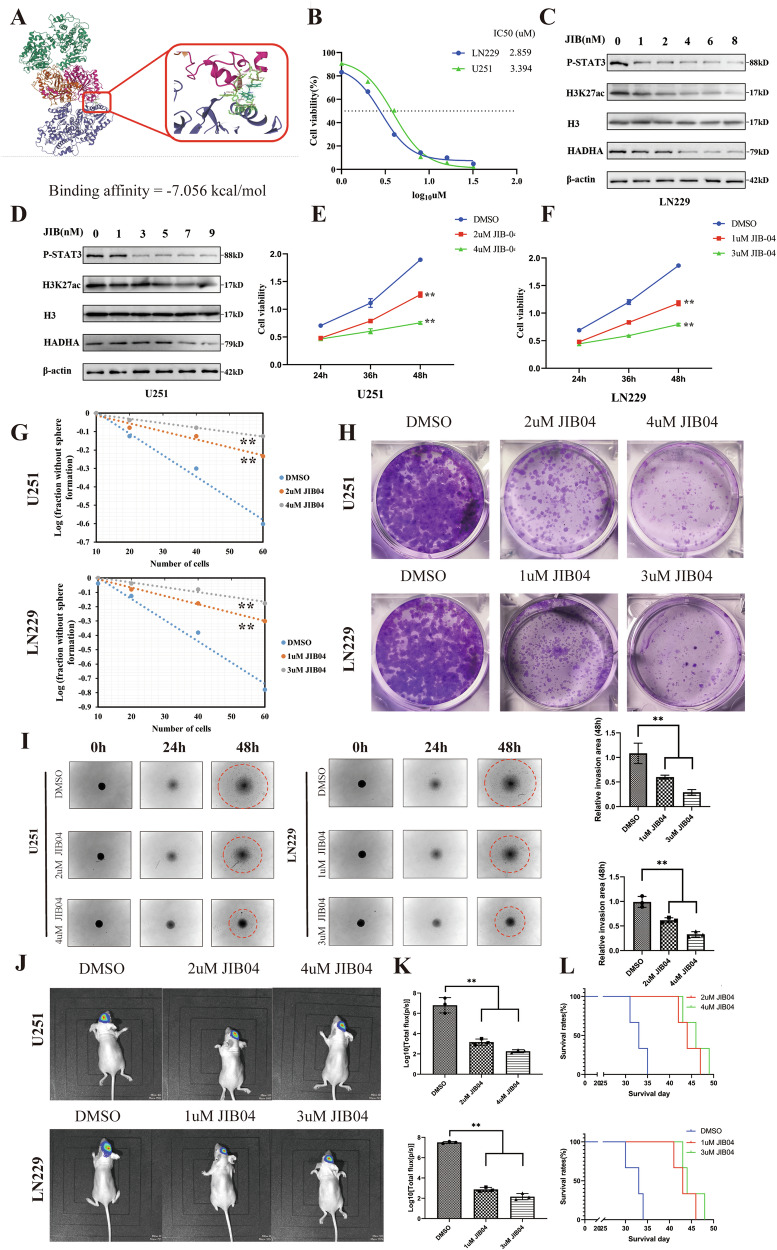
JIB-04 interferes with the HADHA/JAK/STAT axis and is a potential molecular agent for treating GBM. **A** Molecular docking analysis showing the binding affinity of JIB-04 to HADHA with a score of −7.056 kcal/mol, indicating a strong binding interaction. **B** The IC50 of JIB-04 in LN229 and U251 cells. **C**, **D** Western Blot analysis of protein expression in response to JIB-04 treatment in LN229 and U251 Cells. **E**, **F** Dose-dependent effects of JIB-04 on cell viability in U251 and LN229 cells. Cells were treated with increasing concentrations of JIB-04 (2 μM, 4 μM), and cell viability was assessed at 0 h, 48 h, and 24 h. **G** ELDA results indicating the impact of JIB-04 on the clonogenic potential of GBM cells. **H** The treatment with JIB-04 leads to a dose-dependent decrease in colony formation. **I** Cancer Cell Spheroid Invasion Assay results showing the effect of JIB-04 on the invasive capacity of GBM cells. **J** Bioluminescent imaging showing tumor growth in the JIB04 treatment group and the control group. **K** Kaplan-Meier survival curve showing increased overall survival in mice with JIB04 treatment compared to the control group (*P* < 0.05). **L** In vivo tumor growth analysis in a mouse orthotopic model. Tumor volume was significantly smaller in the JIB04 treatment group compared to the control group at day 35 (*P* < 0.001).

JIB-04 is a small-molecule drug we identified that targets HADHA. It is a pan-histone lysine demethylase inhibitor and has also been found to be an effective anticancer agent, capable of combating various cancers, such as hepatocellular carcinoma [10]. Previous studies have shown that JIB-04 has good blood-brain barrier permeability [11]. We investigated whether JIB-04 inhibits HADHA in GBM cells. Molecular docking showed JIB-04 binds to HADHA with a binding energy of -7.056 kcal/mol (Fig. 6A). The IC50 of JIB-04 in LN229 and U251 cells was determined (Fig. 6B), and higher JIB-04 concentrations decreased H3K27ac and P-STAT levels (Fig. 6C, D). JIB-04 significantly inhibited cell proliferation in a dose-dependent manner (Fig. 6E, F). ELDA experiments confirmed its effect on proliferation (Fig. 6G). Additionally, JIB-04 reduced colony formation (Fig. 6H) and inhibited cell invasion in a 3D assay (Fig. 6I). In vivo imaging results showed that JIB04 treatment significantly inhibited tumor growth, reduced tumor volume, and prolonged the overall survival of mice (Fig. 6J–L).

## Discussion

This study provides a comprehensive exploration of the role of HADHA in GBM, revealing its mechanism in activating the JAK/STAT3 signaling pathway through the modulation of H3K27ac histone acetylation. The findings not only confirm the association between the overexpression of HADHA in GBM and poor prognosis but also elucidate the impact of HADHA-mediated metabolic changes on GBM progression, particularly in regulating tumor growth and signal transduction.

Our research further underscores the significance of the intersection between metabolism and epigenetics in oncology. The regulatory effect of HADHA on H3K27ac highlights how metabolic alterations can influence epigenetic marks, potentially affecting gene expression and the functionality of tumor cells. This revelation provides a theoretical foundation for developing new therapeutic strategies that may intervene in epigenetic marks by modulating metabolic pathways, thereby inhibiting tumor development. Recent studies have also emphasized the importance of metabolic enzymes in regulating histone acetylation and gene expression, supporting our findings [3].

The results emphasize the dual role of HADHA, an important metabolic enzyme, in GBM progression: on one hand, it directly influences histone acetylation by regulating acetyl-CoA levels; on the other hand, it indirectly controls the proliferation and survival of tumor cells by affecting the activation status of the JAK/STAT3 signaling pathway. These discoveries offer a new perspective for targeted GBM therapy, suggesting that inhibiting the activity of HADHA or its metabolic pathways could disrupt the malignant behavior of tumors. The role of metabolic enzymes as therapeutic targets in cancer has been previously reported, reinforcing the potential of HADHA as a target [12, 13].

Additionally, our study found that JIB-04 can interfere with HADHA function and inhibit the proliferation and invasion of GBM cells in vitro and in vivo. As a potential therapeutic agent, JIB-04’s interference with the HADHA/JAK/STAT3 axis provides a new molecular approach for GBM treatment. Future research is needed to further explore the pharmacological effects of JIB-04 and assess its potential in clinical GBM therapy. Studies on the efficacy of JIB-04 in other cancer types have shown promising results, which may be relevant to its application in GBM [10, 14, 15].

It is worth noting that, although JIB-04 has been shown to interfere with HADHA function in this study, detailed data regarding the specificity of JIB-04 for HADHA have not yet been provided. JIB-04 also acts on histone methylation. This fact suggests that the effects of HADHA inhibition observed in our study may also be attributed to changes in histone methylation, rather than merely acetylation. Like acetylation, histone methylation is a key epigenetic modification that can significantly impact gene expression and cellular function. Future studies should investigate the specific effects of HADHA inhibition on histone methylation marks and explore whether histone methylation and acetylation interact in the context of HADHA inhibition to collectively influence the phenotype of GBM cells.

Although this study offers valuable insights into the role of HADHA in GBM, additional research is needed to validate these findings and investigate the role of HADHA in other cancer types. Moreover, future studies should focus on developing new therapeutic strategies that may combine metabolic modulators and epigenetic modifiers to more effectively treat GBM. In conclusion, this research offers new perspectives for understanding the complex biology of GBM and lays the foundation for developing new therapeutic strategies.

By delving deeper into the interactions between metabolism, epigenetics, and signaling pathways, we hope to provide more effective treatment options for GBM patients. Through a comprehensive understanding of these complex biological processes, we can potentially develop targeted therapies that disrupt the metabolic-epigenetic axis, offering new hope for the treatment of GBM. Recent advances in the understanding of metabolic-epigenetic crosstalk in cancer have opened up new avenues for therapeutic intervention, which may be leveraged in the future development of GBM treatments [16, 17].

## Materials and methods

### Ethics statement and clinical glioma tumor specimens

The research protocol was reviewed and approved by the Ethics Committee of the Fourth Affiliated Hospital of Harbin Medical University (Ethical approval number: 2023-LLSC-50). Written informed consent was obtained from each participant prior to enrollment. Glioma tumor specimens were collected during surgical procedures. Additionally, non-neoplastic brain tissues were obtained from individuals undergoing surgery for traumatic brain injuries as control samples.

### Data processing and bioinformatics analysis

In this study, we obtained genomic and transcriptomic data of GBM patients from the CGGA database and TCGA. To comprehensively understand the expression of HADHA in the nervous system, we utilized the GETx online analysis tool, which can display the expression patterns of specific genes in different brain regions and neurological diseases. Additionally, we employed the SingerBox online tool to analyze the mutation frequency of HADHA, assessing its potential genomic instability in GBM. We also leveraged the LinkedOmicsKB online platform to explore the correlations between HADHA mRNA and various phenotypes, including the activation status of the JAK/STAT3 pathway and the infiltration of M1 and M2 macrophages.

### Cell culture and transfection

NHA, LN229, U87, A172, T98G, and U251 cell lines were obtained from Procell Life Science & Technology (Wuhan, China) and cultured at 37 °C in DMEM (Haixing Biosciences, China) containing 10% FBS and 5% CO2. HADHA-shRNA lentivirus was obtained from Genechem (Shanghai, China). For transfection with HADHA-shRNA, cells were cultured to the logarithmic growth phase and adjusted to a density of 50–60%. The HADHA-shRNA lentivirus was added to the cell culture, incubated for 4–6 h, and then replaced with fresh medium containing 10% FBS for 48–72 h before analysis.

### qRT-PCR analysis

Total RNA was extracted using TRIzol reagent (Thermo Fisher Scientific, USA), and 1 μg of RNA was reverse-transcribed into cDNA using a reverse transcription kit (Seven, Beijing, China). qRT-PCR was performed with SYBR Green master mix (Seven, Beijing, China) on a StepOnePlus system (Applied Biosystems, USA). The thermal cycling included 95 °C for 3 min, then 40 cycles of 95 °C for 15 s and 60 °C for 30 s. Gene expression was normalized to β-Actin using the ΔΔCt method. All reactions were performed in triplicate. Primer details are in Supplementary Table 1.

### Western blot analysis

Proteins were extracted using RIPA buffer (Servicebio, China) with protease and phosphatase inhibitors. Protein concentrations were measured using the BCA Protein Assay Kit (Thermo Fisher Scientific, USA). Equal protein amounts (20–40 μg) were separated on 10% SDS-PAGE gels and transferred to PVDF membranes (Millipore, USA). Membranes were blocked with 5% non-fat milk in TBST for 1 h, incubated overnight at 4 °C with primary antibodies (Supplementary Table 2) and Actin (1:1000, Bioss, China) as a loading control. After washing, membranes were incubated with HRP-conjugated secondary antibodies (1:5000, Beyotime, China) for 1 h. Protein bands were detected using an ECL kit (HUABIO, China) and imaged. Band intensities were quantified with ImageJ software and normalized to ACTIN. All experiments were performed in triplicate.

### Cell viability assay

Cells were plated in 96-well plates at 2 × 10³ cells per well and cultured in DMEM. After treatment, cells were incubated for 24 or 48 h, then 10 μl of CCK-8 reagent (Beyotime, China) was added. After 1 h of incubation at 37 °C, cell viability was measured at 450 nm using a microplate reader. Experiments were performed in triplicate.

### Colony formation assay

Cells were seeded at 500 cells/well in 6-well plates and cultured for 10–14 days. Following PBS washing, they were fixed with 4% paraformaldehyde for 15 min and stained with 0.5% crystal violet (Solarbio, China) for 30 min. Colonies containing over 50 cells were counted microscopically. The experiment was conducted in triplicate.

### Extreme limiting dilution assays (ELDA)

Firstly, cultivate the U251 and LN229 cell lines in a suitable medium until they reach the logarithmic phase of growth. Next, perform a serial dilution of the cell suspension to ensure that the number of cells per well is close to a single-cell level. Then, seed the diluted cell suspension into a 96-well plate, adding 0.1 mL of cell suspension per well. Regularly observe the growth of cells in each well under a microscope, particularly at the end of the culture period, and record the number of wells in which tumor spheres have formed. Conduct statistical analysis on the experimental data to compare the differences between the drug-treated groups at various concentrations and the control group.

### Cancer cell spheroid invasion assay

Spheroids of U251 and LN229 cells (500 cells per droplet) were formed using the hanging drop technique on the lids of culture dishes. After 48 h of incubation, the spheroids were harvested and embedded in rat tail type I collagen to establish a 3D culture system. The extent of invasion was evaluated 3 days post-embedding by analyzing the maximum invasion distance and invaded area with ImageJ software.

### Flow cytometric analysis

Cells were collected, washed with ice-cold PBS, and fixed overnight in 70% ethanol at 4 °C. After fixation, the cells were stained with propidium iodide (PI) reagent (Beyotime, Shanghai, China) and incubated in the dark at 37 °C for 30 min. Red fluorescence was measured at 488 nm excitation using a flow cytometer, and light scattering was recorded. Cell cycle distribution was analyzed using FlowJo software.

### Immunohistochemistry

Tissue sections were fixed in 4% paraformaldehyde, embedded in paraffin, and cut into 4 μm slices. After baking at 60 °C, sections were deparaffinized, rehydrated, and subjected to antigen retrieval. Endogenous peroxidase was blocked with 3% hydrogen peroxide, and non-specific binding with 5% goat serum. Sections were incubated with primary antibodies overnight, followed by HRP-conjugated secondary antibodies. Staining was visualized with DAB and counterstained with hematoxylin, then analyzed with ImageJ

### Acetyl-CoA measurement

Cells or tissue samples were homogenized in ice-cold PBS or lysis buffer, followed by centrifugation to remove debris. The supernatant was collected for analysis. Acetyl-CoA levels were measured using an Acetyl-CoA Assay Kit (Elabscience, China). Acetyl-CoA reacts with a substrate and enzyme mix, generating a colorimetric or fluorometric signal proportional to the concentration. A standard curve was prepared with known acetyl-CoA concentrations. Absorbance or fluorescence was measured, and acetyl-CoA concentration was calculated from the standard curve.

### ChIP-qPCR

Cells were crosslinked with 1% formaldehyde for 10–15 min, then quenched with 0.125 M glycine for 5 min. After washing with ice-cold PBS, cells were lysed in buffer with protease inhibitors. Chromatin was sheared by sonication into 200–1000 bp fragments and clarified by centrifugation. The supernatant was used for immunoprecipitation. After pre-clearing with protein A/G beads, chromatin was incubated overnight with anti-H3K27ac antibody at 4 °C. Protein A/G beads captured the antibody-bound chromatin, which was washed and eluted. After reverse crosslinking at 65 °C and DNA purification, qPCR was performed using specific primers (Supplementary Table 3), and target DNA enrichment was calculated by the ΔΔCT method.

### Molecular docking

Molecular docking analysis was performed with AutoDock Vina 1.2.2 to assess the binding affinity and interaction of the drug candidate with its targets. The ligand structure was obtained from PubChem, and the HADHA protein (PDB ID: 5ZQZ) from the Protein Data Bank. Both files were converted to PDBQT format, excluding water molecules and adding polar hydrogen atoms. The docking grid, with a size of 30 Å × 30 Å × 30 Å, was centered on the HADHA active site. All simulations were run using AutoDock Vina 1.2.2.

### Orthotopic xenograft mouse model of glioblastoma

To investigate the role of HADHA in tumor progression, this experiment used two GBM cell lines. The cells were first cultured and expanded to the logarithmic phase. Then, the cells were harvested by trypsinization, neutralized with PBS, and counted using a hemocytometer or an automated cell counter. The cell suspension was adjusted to the required concentration. Next, the modified GBM cell suspension (~1 × 10^6^ to 1 × 10⁷ cells/5 μl) was injected the brain of nude mice by drilling a small hole in the skull. The nude mice used in the experiment were purchased from Jiangsu Huachuang Sino Pharma Tech Co., Ltd. Each nude mouse was injected with 5 μl of the cell suspension to ensure even distribution. After the surgery, the health of the nude mice was regularly monitored, including body weight, activity level, and any signs of distress or tumor-related symptoms. To assess tumor growth, optical imaging techniques were used to evaluate the tumor volume and location. At the end of the experiment (typically after 4–6 weeks), when the nude mice showed significant weight loss or health issues, they were euthanized. Brain tissue was harvested, and tumor size and weight were measured. Immunohistochemistry or other histological methods were employed for further analysis. All animal experiments followed ethical guidelines and were approved by the Institutional Animal Care and Use Committee.

### Statistical analysis

The study adhered to randomized and blinded experimental protocols throughout all procedures. Three independent experiments were conducted, and the experimental results were shown as mean ± standard deviation. Data calculations and statistical analyses were conducted using R software (version 4.0.2). For continuous variables, Student’s *t*-test was used for normal distribution and the Mann-Whitney *U* test for non-normal distribution. Statistical significance was set at *P* < 0.05.

## Supplementary information

Figure S1

supplementary figure 1 legend

WB raw data

Supplementary Table 1

Supplementary Table 2

Supplementary Table 3

## Data Availability

The data supporting the findings of this study are available from the corresponding author upon reasonable request.
