# Supplementary figures and images for: HADHA-mediated regulation of JAK/STAT3 signaling in glioblastoma: a metabolic-epigenetic axis
Source: Cell Death Discov. 2025 Aug 1;11:361. doi: 10.1038/s41420-025-02660-0 (PMC12316893; doi:10.1038/s41420-025-02660-0)

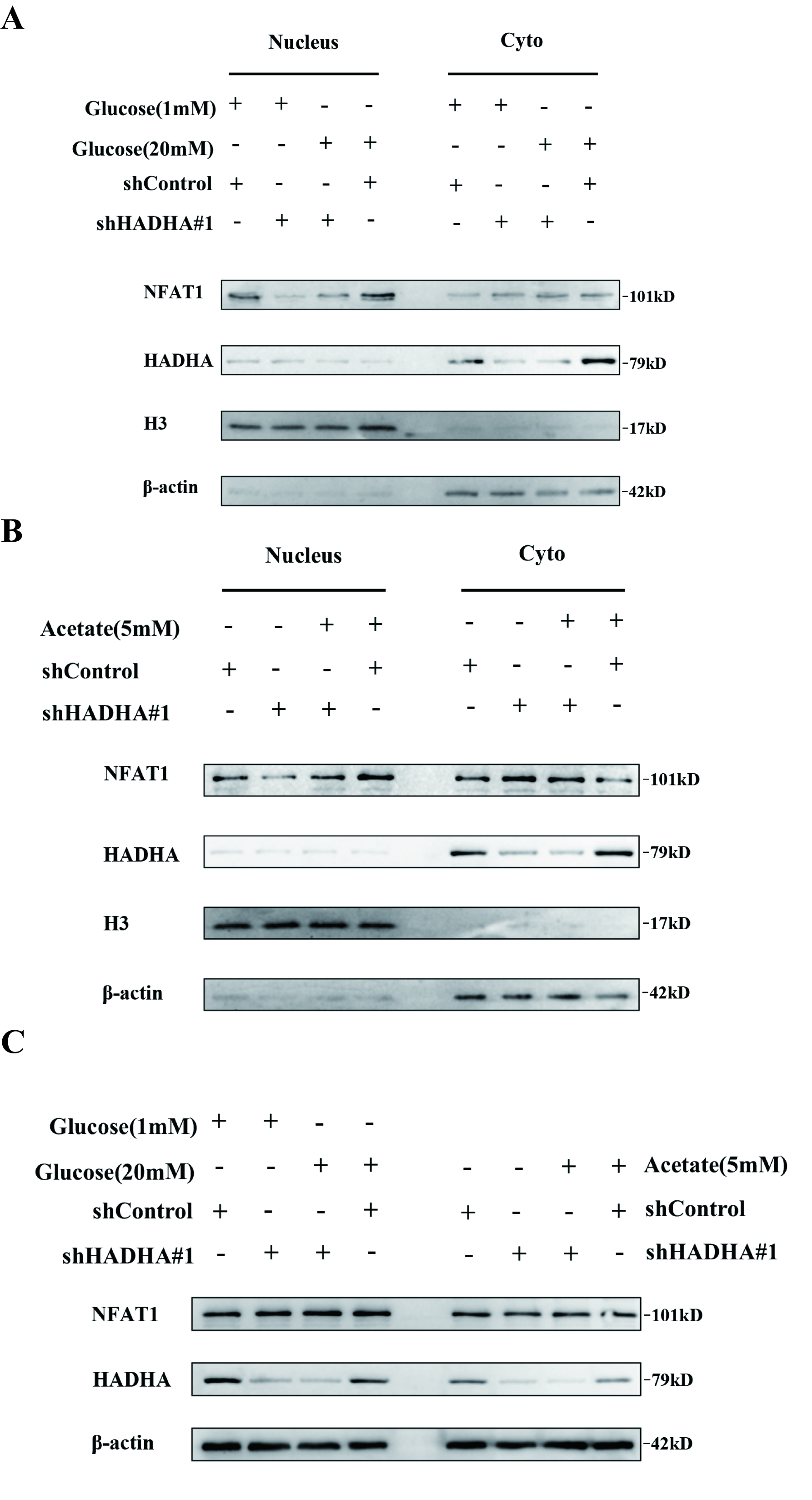

Supplement: Supplementary file 1 — Figure S1 [file 41420_2025_2660_MOESM1_ESM.tif]
