# Supplementary material for: HADHA-mediated regulation of JAK/STAT3 signaling in glioblastoma: a metabolic-epigenetic axis
Source: Cell Death Discov. 2025 Aug 1;11:361. doi: 10.1038/s41420-025-02660-0 (PMC12316893; doi:10.1038/s41420-025-02660-0)
Supplement: Supplementary file 2 — supplementary figure 1 legend [file 41420_2025_2660_MOESM2_ESM.docx]

**Figure S1: The Correlation Between Metabolite Supplementation and NFAT1 Nuclear Accumulation**

(A-B) Supplementation with glucose and acetate restored NFAT1 levels in the nucleus. (C) Glucose and acetate did not affect the total NFAT1 expression.
