# Supplementary material for: HADHA-mediated regulation of JAK/STAT3 signaling in glioblastoma: a metabolic-epigenetic axis
Source: Cell Death Discov. 2025 Aug 1;11:361. doi: 10.1038/s41420-025-02660-0 (PMC12316893; doi:10.1038/s41420-025-02660-0)
Supplement: Supplementary file 3 — WB raw data [file 41420_2025_2660_MOESM3_ESM.pdf]

Figure 1H

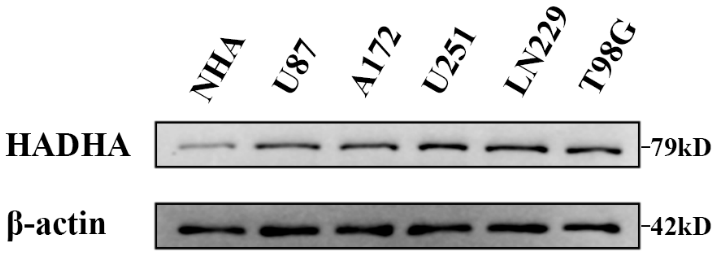

NHA
U87
A172
U251
LN229
T98G
HADHA
-79kD
β-actin
-42kD

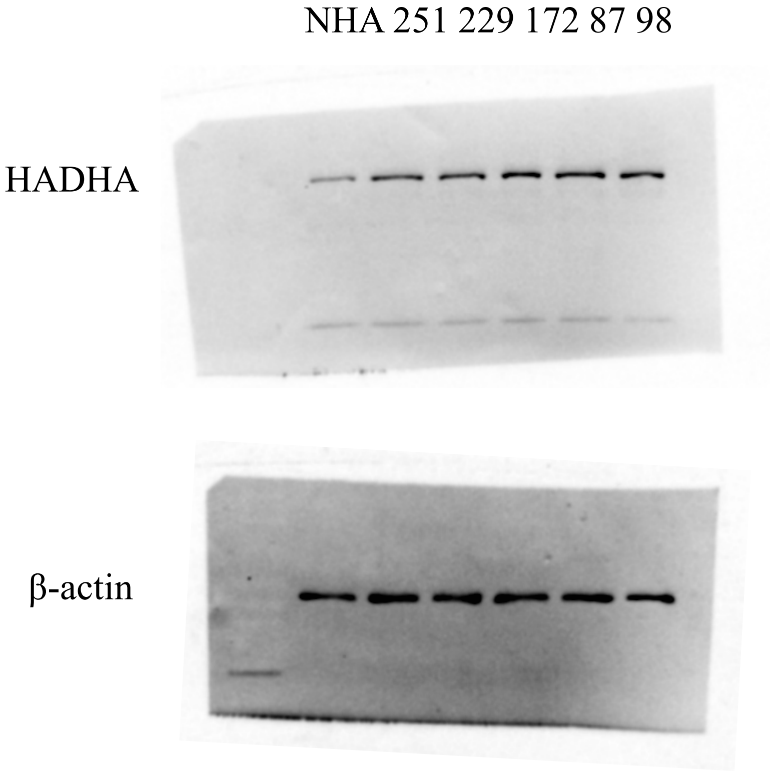

NHA 251 229 172 87 98
HADHA
β-actin

Figure 2A

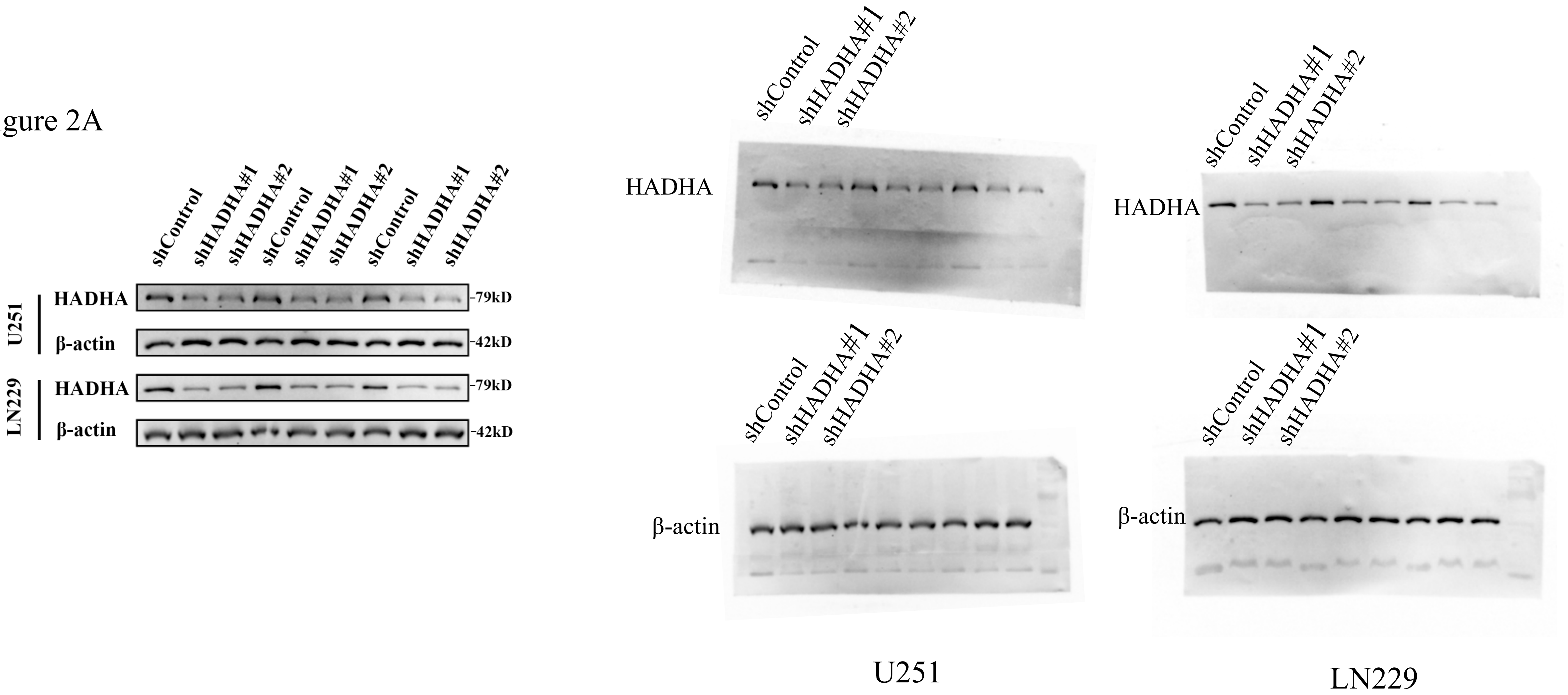
shControl
shHADHA#1
shHADHA#2
U251
LN229
HADHA
β-actin
-79kD
-42kD

Figure 3C

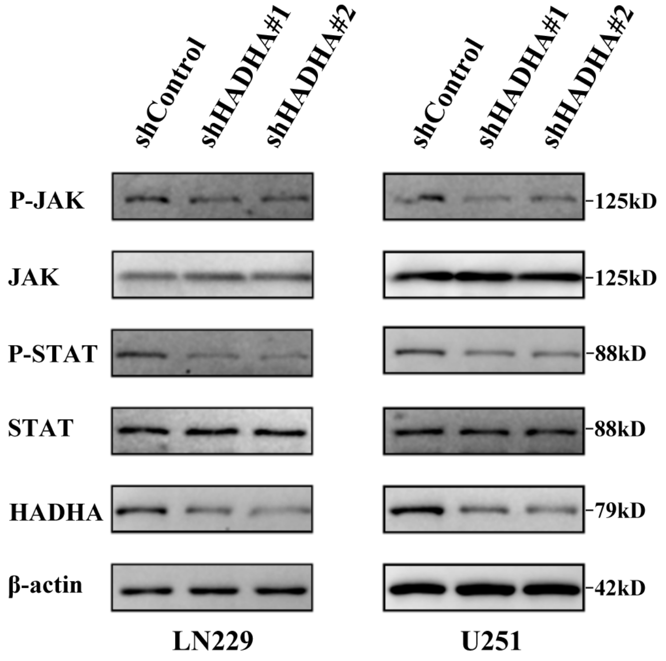

shControl
shHADHA#1
shHADHA#2
shControl
shHADHA#1
shHADHA#2
P-JAK
JAK
P-STAT
STAT
HADHA
β-actin
-125kD
-125kD
-88kD
-88kD
-79kD
-42kD
LN229
U251

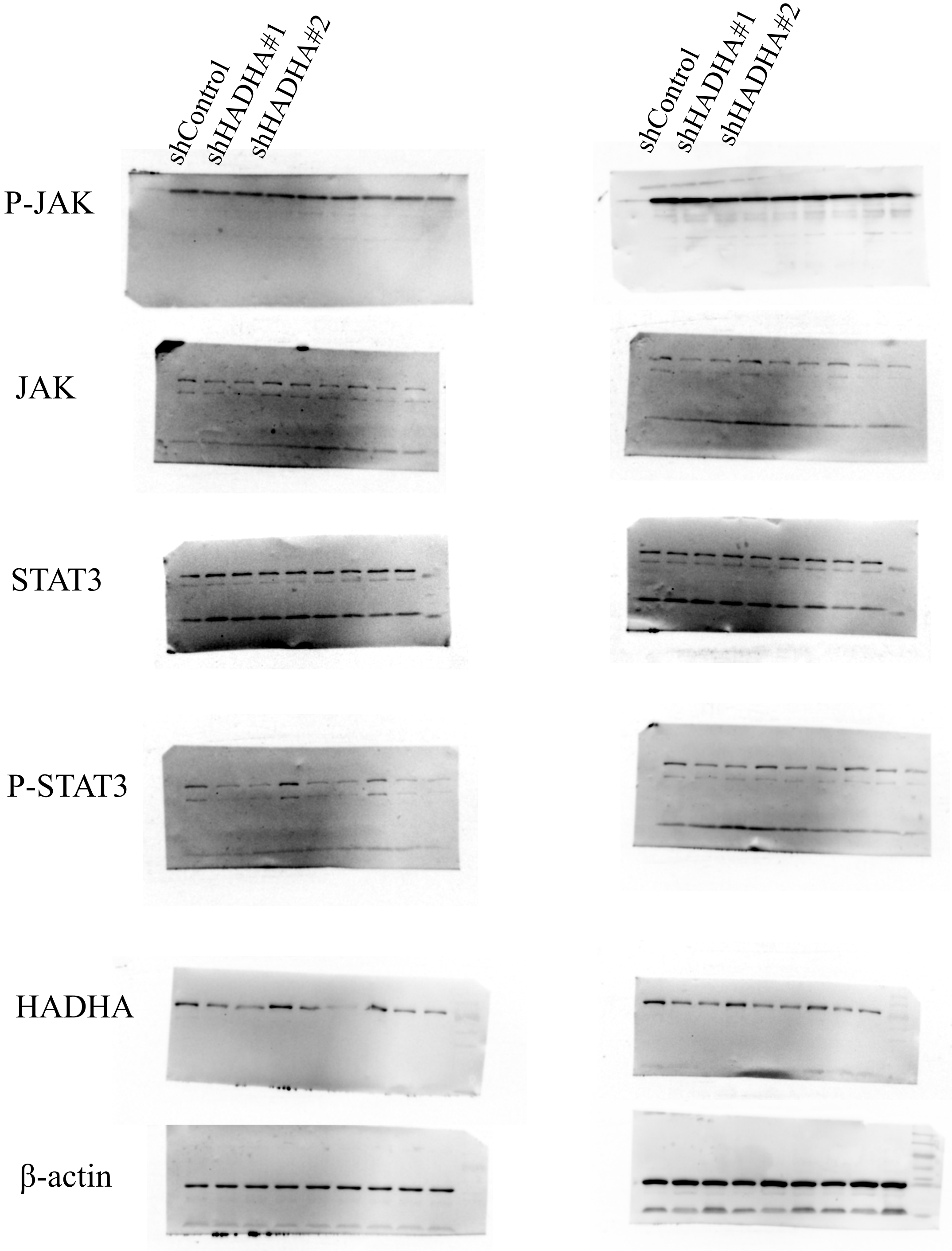

shControl
shHADHA#1
shHADHA#2
shControl
shHADHA#1
shHADHA#2
P-JAK
JAK
STAT3
P-STAT3
HADHA
β-actin

Figure 3D

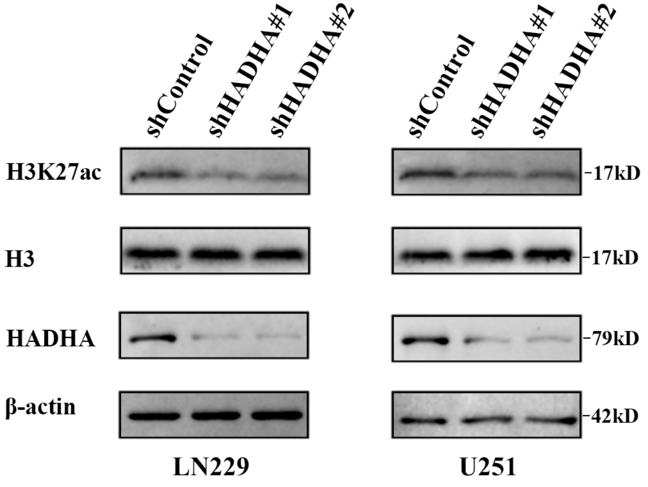
shControl
shHADHA#1
shHADHA#2
shControl
shHADHA#1
shHADHA#2
H3K27ac
H3
HADHA
β-actin
-17kD
-17kD
-79kD
-42kD
LN229
U251

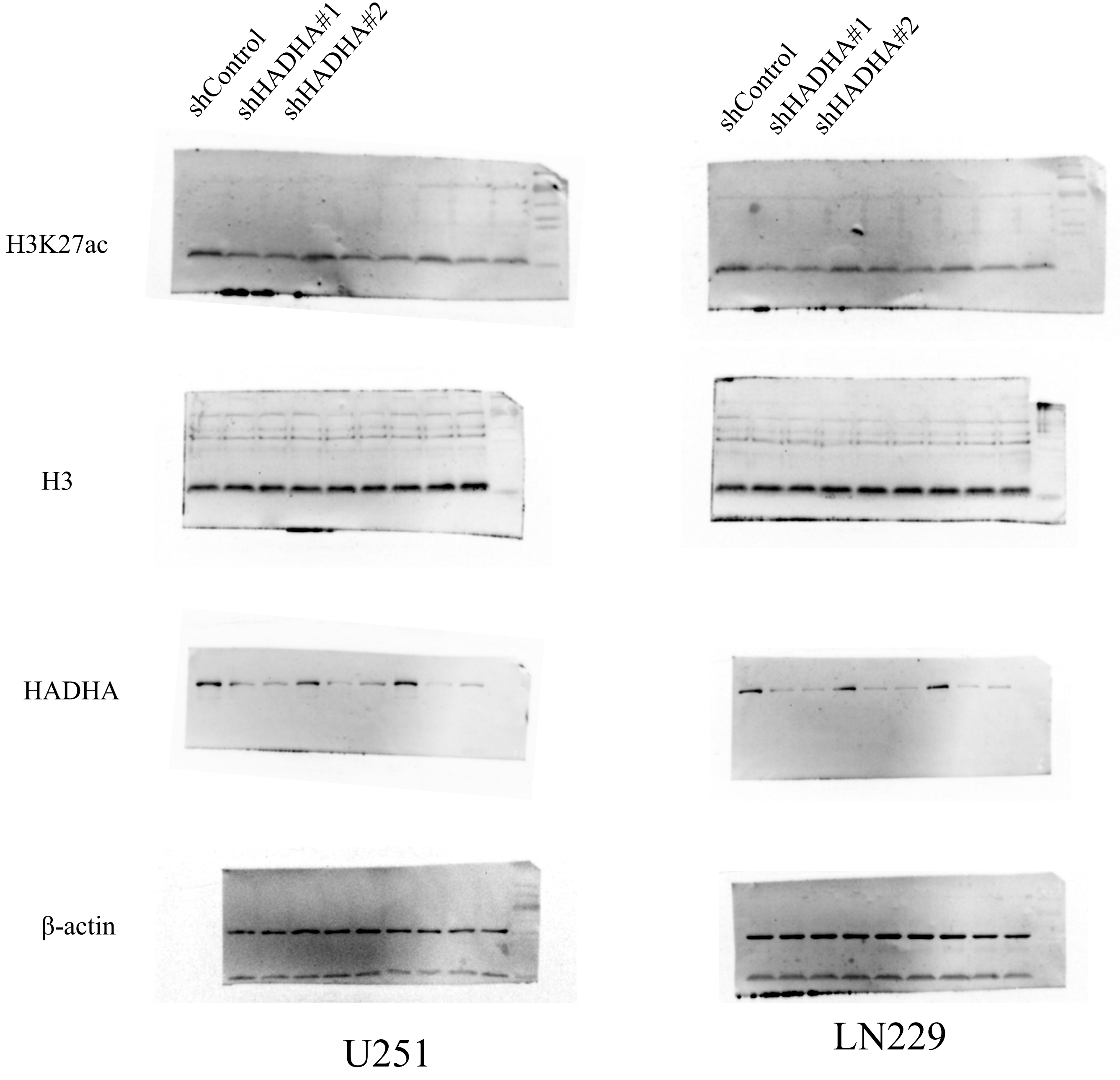
shControl
shHADHA#1
shHADHA#2
shControl
shHADHA#1
shHADHA#2
H3K27ac
H3
HADHA
β-actin
U251
LN229

Figure 3E

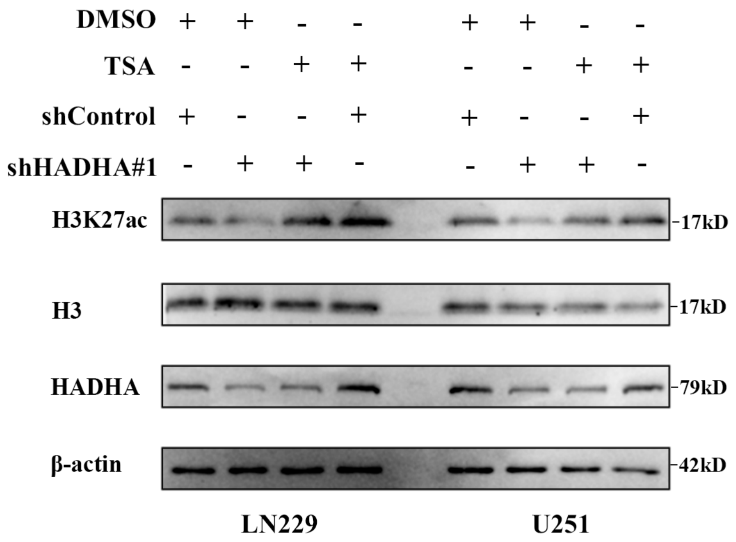
DMSO + + - - + + - -
TSA - - + + - - + +
shControl + - - + + - - +
shHADHA#1 - + + - - + + -
H3K27ac
-17kD
H3
-17kD
HADHA
-79kD
β-actin
-42kD
LN229
U251

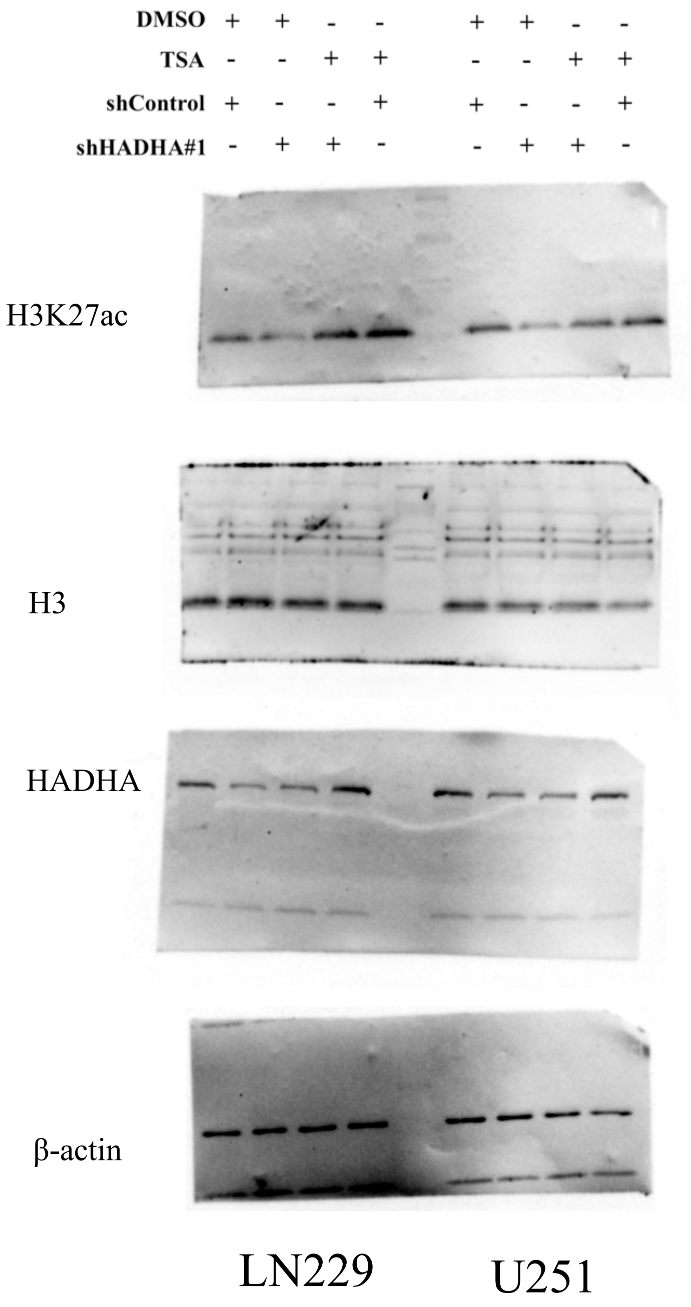
DMSO + + - - + + - -
TSA - - + + - - + +
shControl + - - + + - - +
shHADHA#1 - + + - - + + -
H3K27ac
H3
HADHA
β-actin
LN229
U251

Figure 3F

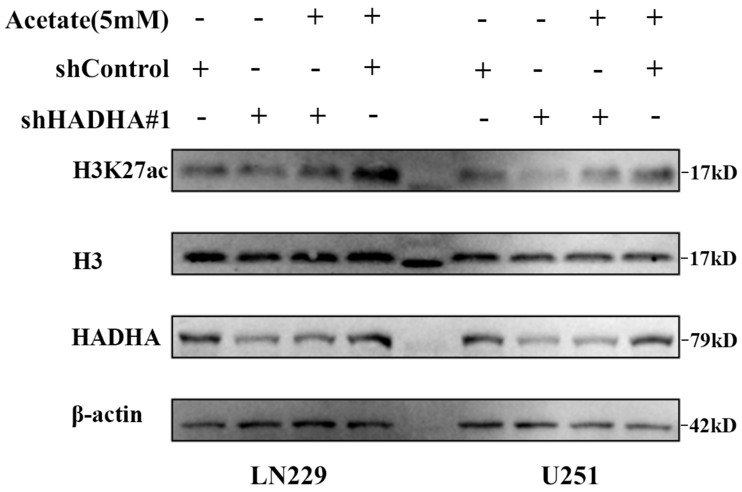

Acetate(5mM) - - + + - - + +
shControl + - - + + - - +
shHADHA#1 - + + - - + + -
H3K27ac
-17kD
H3
-17kD
HADHA
-79kD
β-actin
-42kD
LN229
U251

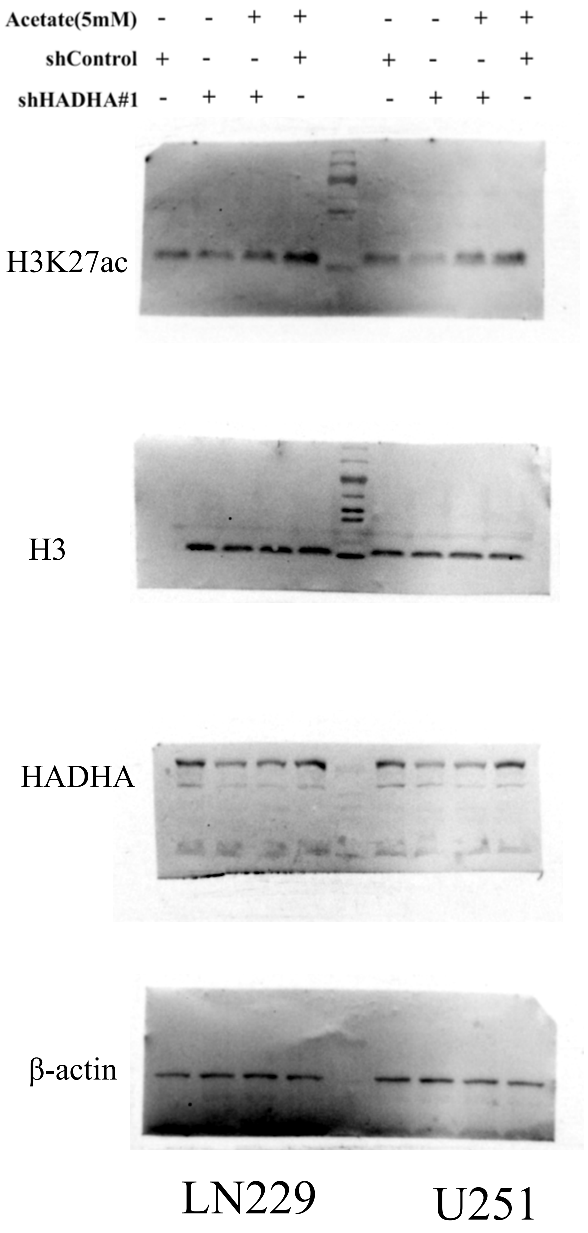

Acetate(5mM) - - + + - - + +
shControl + - - + + - - +
shHADHA#1 - + + - - + + -
H3K27ac
H3
HADHA
β-actin
LN229
U251

Figure 3H

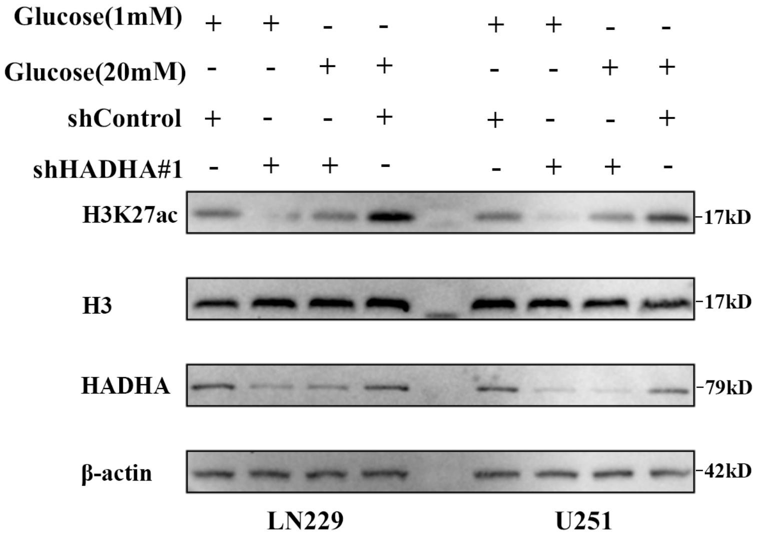
Glucose(1mM) + + - - + + - -
Glucose(20mM) - - + + - - + +
shControl + - - + + - - +
shHADHA#1 - + + - - + + -
H3K27ac
H3
HADHA
β-actin
17kD
17kD
79kD
42kD
LN229
U251

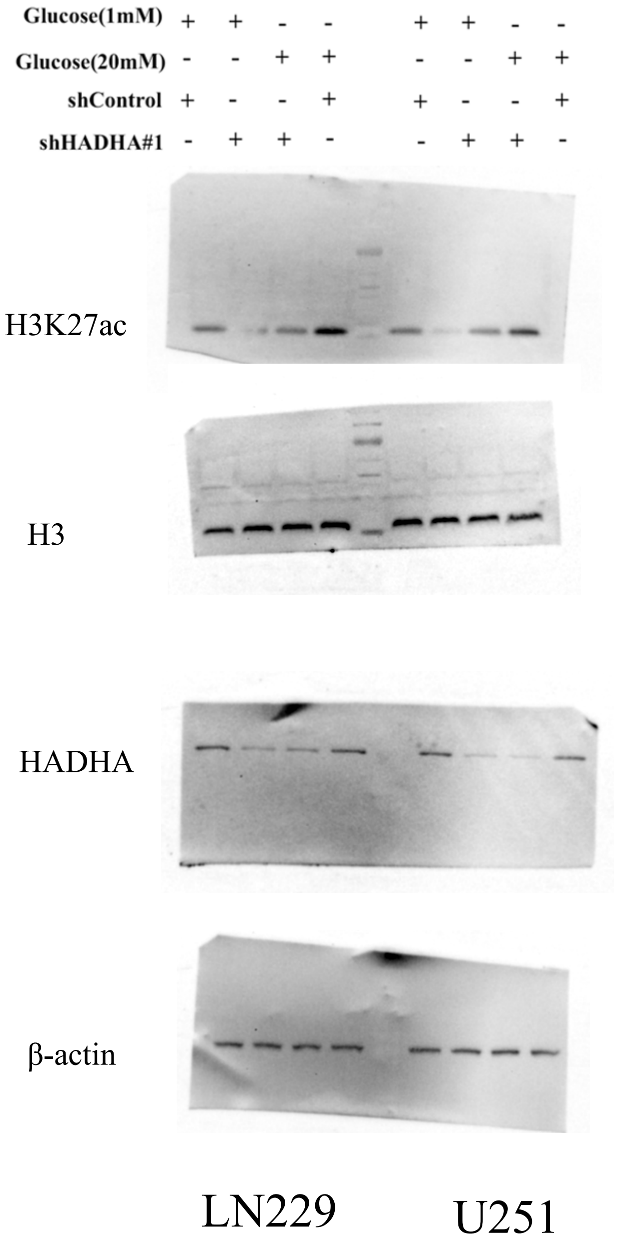
Glucose(1mM) + + - - + + - -
Glucose(20mM) - - + + - - + +
shControl + - - + + - - +
shHADHA#1 - + + - - + + -
H3K27ac
H3
HADHA
β-actin
LN229
U251

Figure 4A

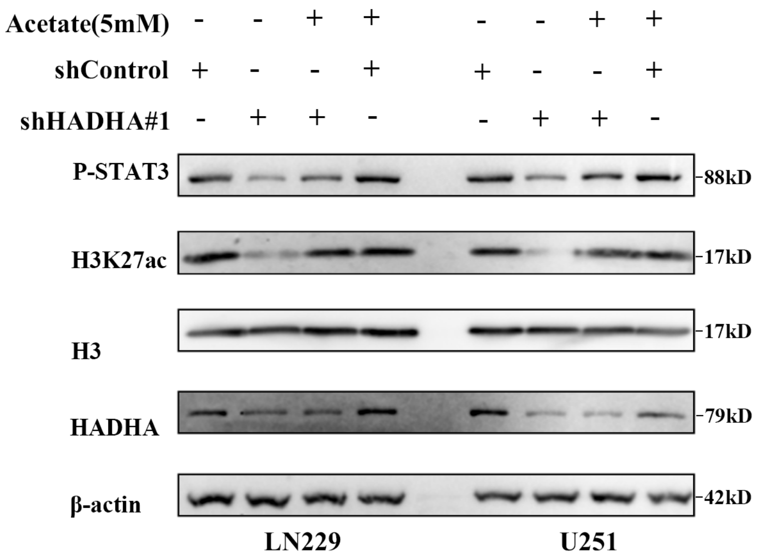

Acetate(5mM) - - + + - - + +
shControl + - - + + - - +
shHADHA#1 - + + - - + + -
P-STAT3
88kD
H3K27ac
17kD
H3
17kD
HADHA
79kD
β-actin
42kD
LN229
U251

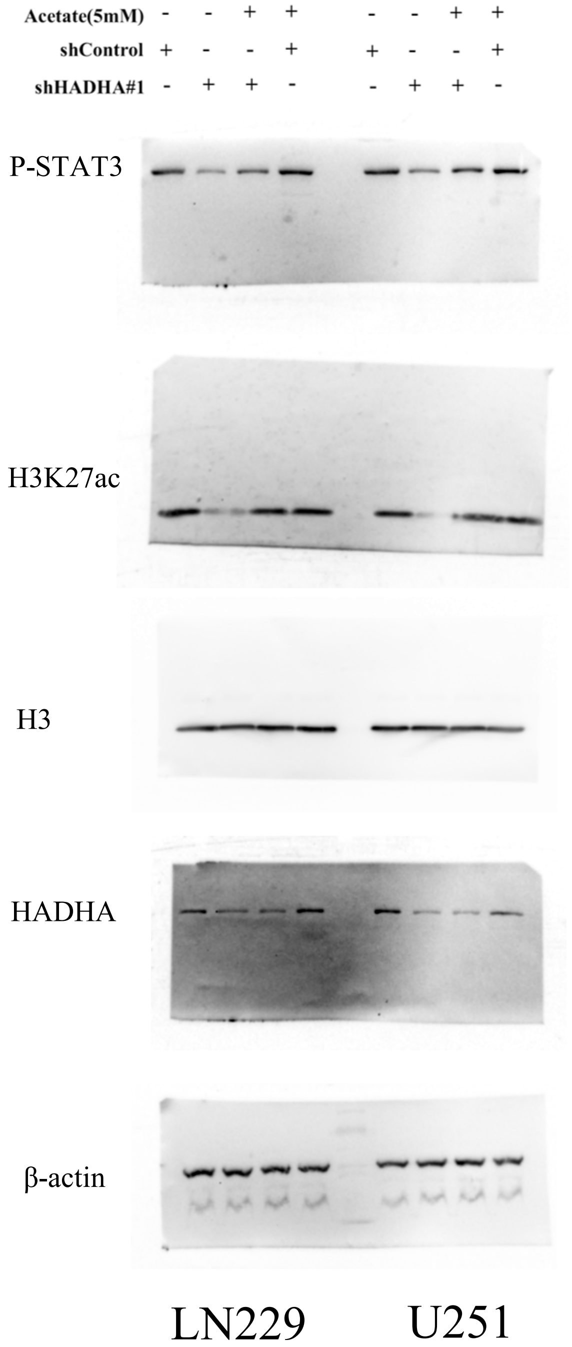

Acetate(5mM) - - + + - - + +
shControl + - - + + - - +
shHADHA#1 - + + - - + + -
P-STAT3
H3K27ac
H3
HADHA
β-actin
LN229
U251

Figure 4B

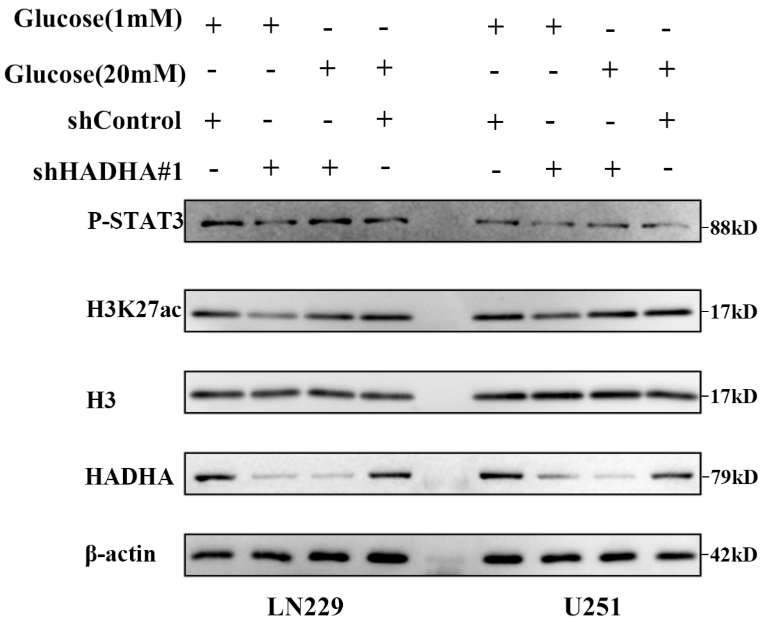
Glucose(1mM) + + - - + + - -
Glucose(20mM) - - + + - - + +
shControl + - - + + - - +
shHADHA#1 - + + - - + + -
P-STAT3
88kD
H3K27ac
17kD
H3
17kD
HADHA
79kD
β-actin
42kD
LN229
U251

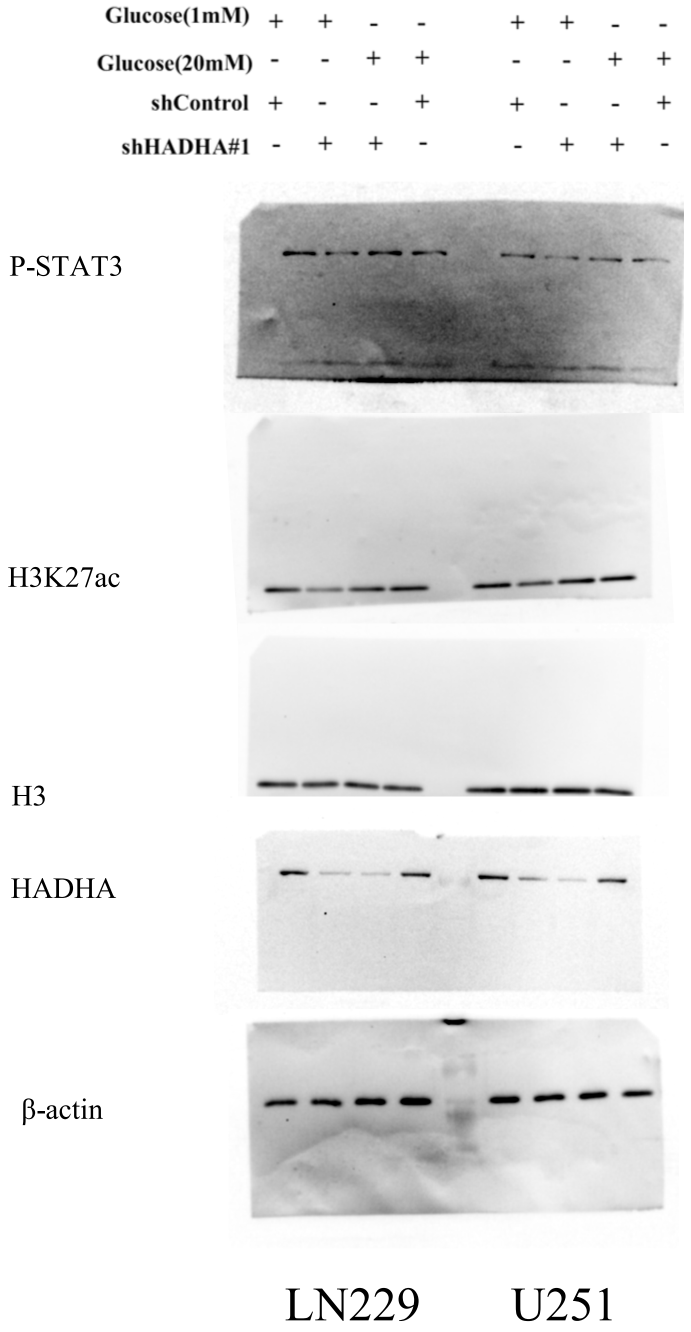
Glucose(1mM) + + - - + + - -
Glucose(20mM) - - + + - - + +
shControl + - - + + - - +
shHADHA#1 - + + - - + + -
P-STAT3
H3K27ac
H3
HADHA
β-actin
LN229
U251

Figure 4E

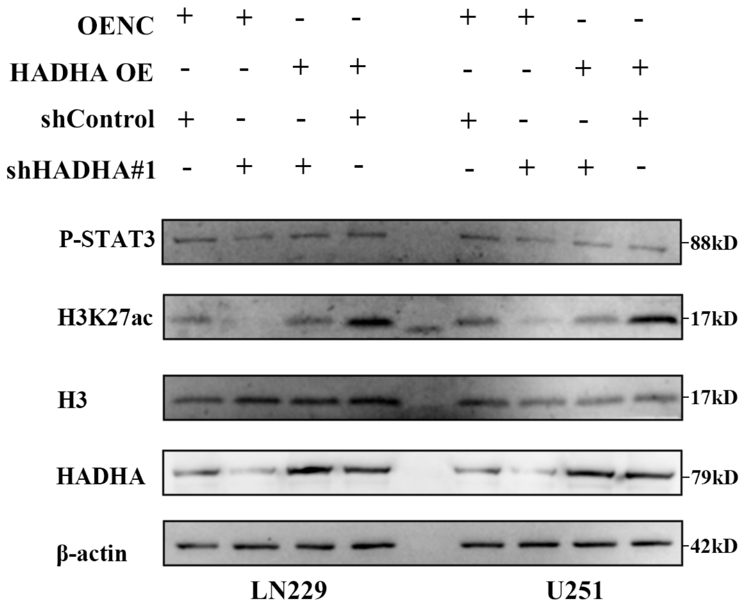
OENC + + - - + + - -
HADHA OE - - + + - - + +
shControl + - - + + - - +
shHADHA#1 - + + - - + + -
P-STAT3
88kD
H3K27ac
17kD
H3
17kD
HADHA
79kD
β-actin
42kD
LN229
U251

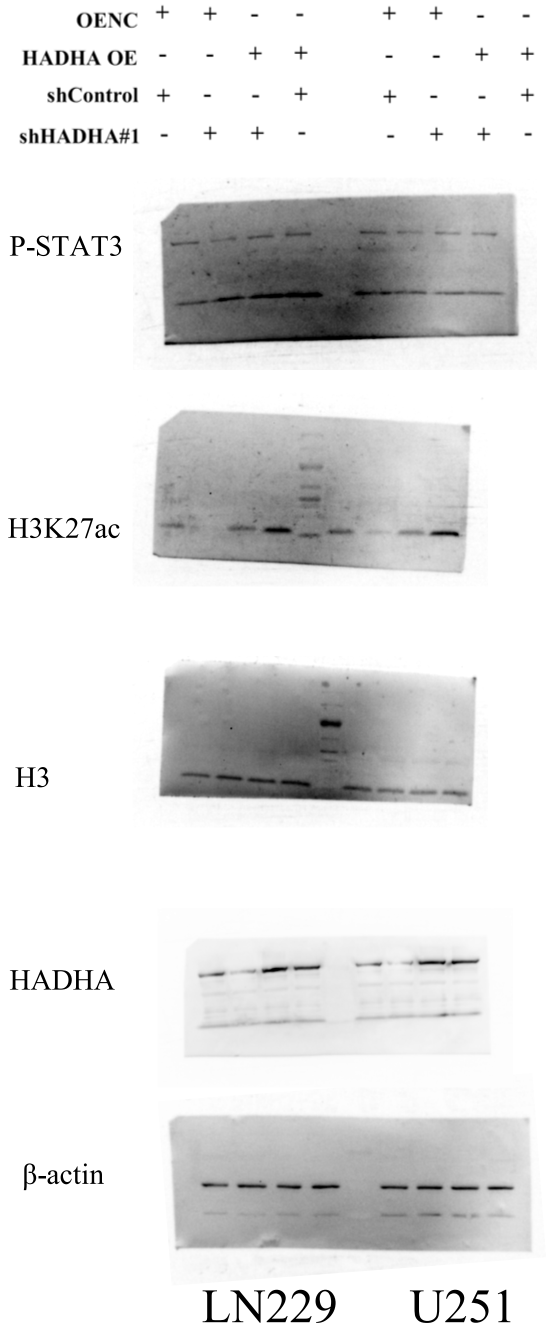
OENC + + - - + + - -
HADHA OE - - + + - - + +
shControl + - - + + - - +
shHADHA#1 - + + - - + + -
P-STAT3
H3K27ac
H3
HADHA
β-actin
LN229
U251

Figure 5A

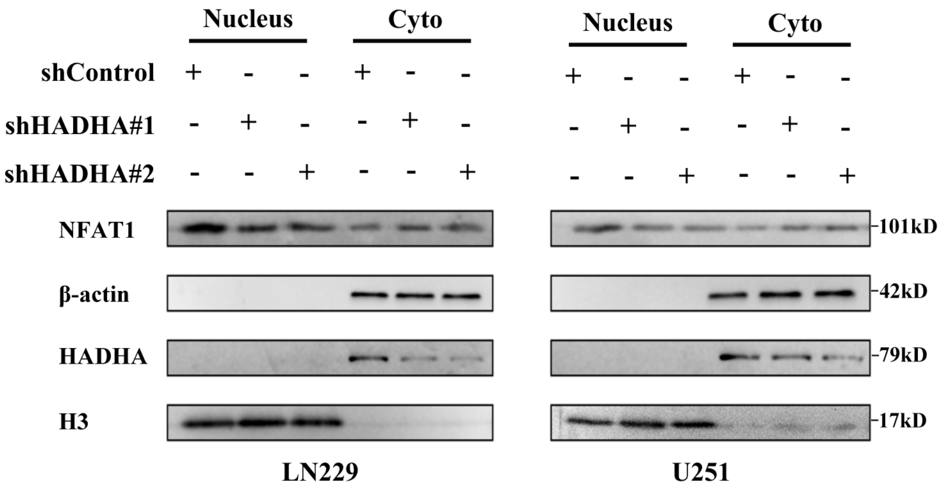

Nucleus
Cyto
Nucleus
Cyto
shControl
shHADHA#1
shHADHA#2
NFAT1
β-actin
HADHA
H3
101kD
42kD
79kD
17kD
LN229
U251

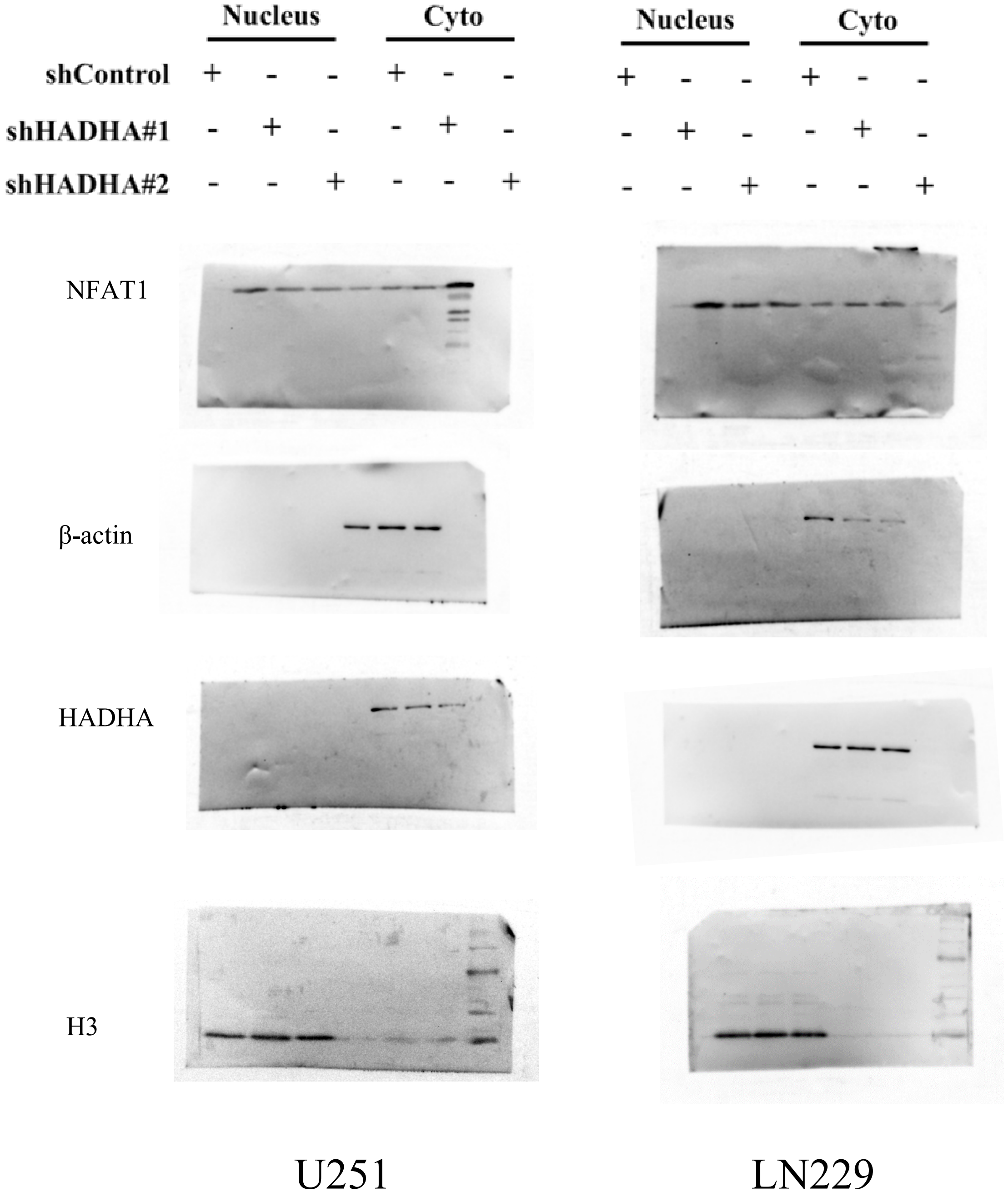

Nucleus
Cyto
Nucleus
Cyto
shControl
shHADHA#1
shHADHA#2
NFAT1
β-actin
HADHA
H3
U251
LN229

Figure 5B

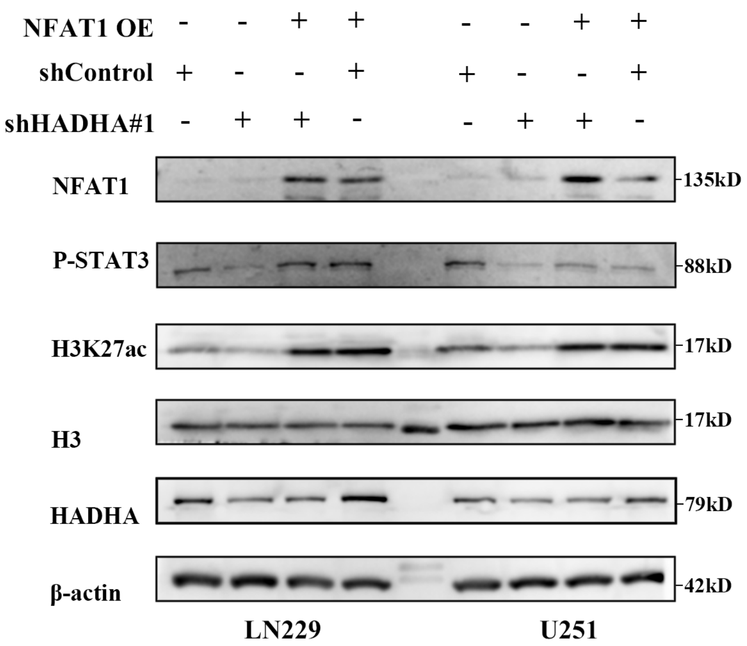
NFAT1 OE - - + + - - + +
shControl + - - + + - - +
shHADHA#1 - + + - - + + -
NFAT1
135kD
P-STAT3
88kD
H3K27ac
17kD
H3
17kD
HADHA
79kD
β-actin
42kD
LN229
U251

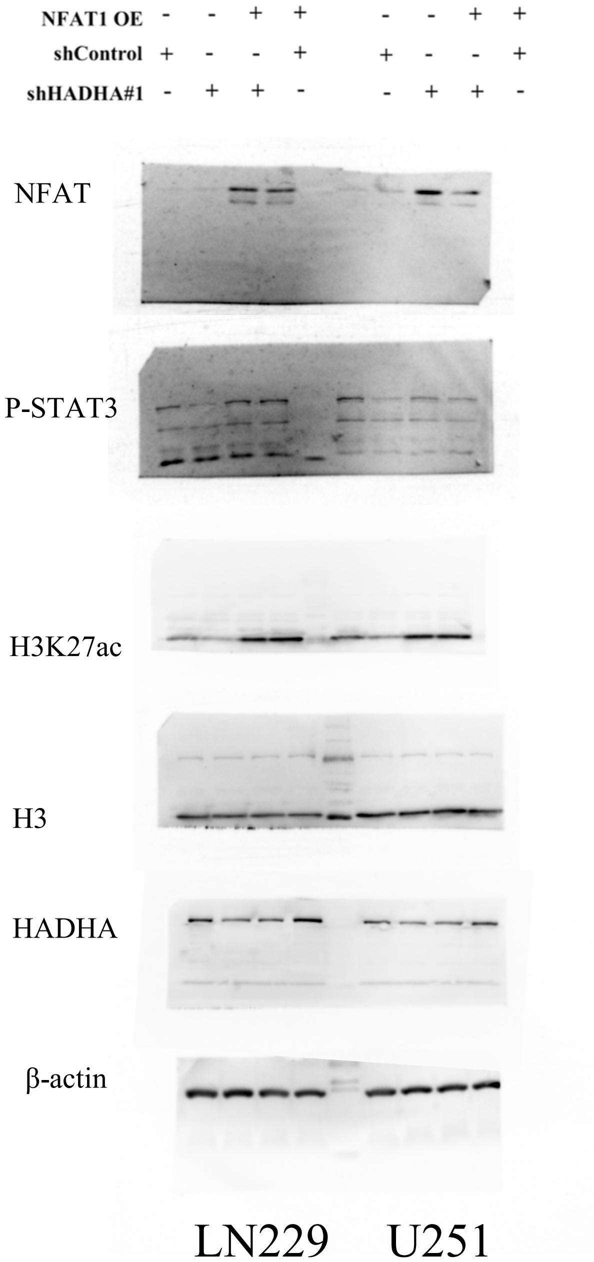
NFAT1 OE - - + + - - + +
shControl + - - + + - - +
shHADHA#1 - + + - - + + -
NFAT
P-STAT3
H3K27ac
H3
HADHA
β-actin
LN229
U251

Figure 6C

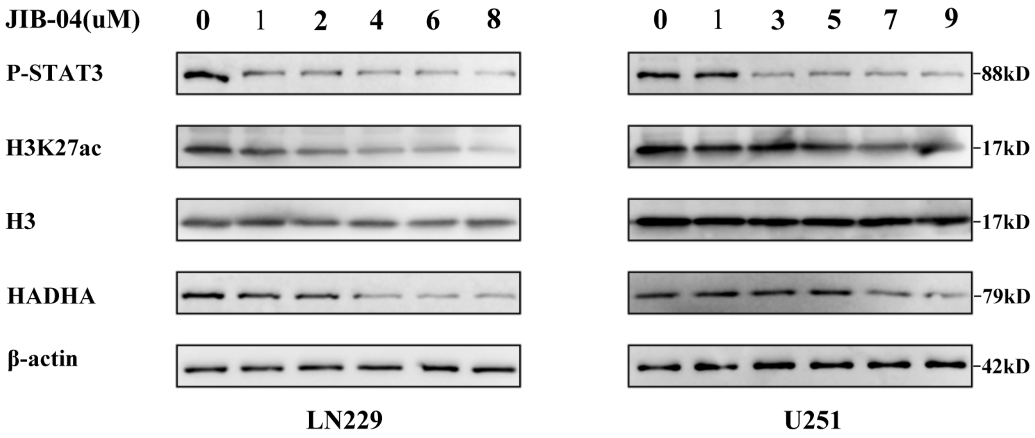

JIB-04(uM)
0
1
2
4
6
8
0
1
3
5
7
9
P-STAT3
H3K27ac
H3
HADHA
β-actin
-88kD
-17kD
-17kD
-79kD
-42kD
LN229
U251

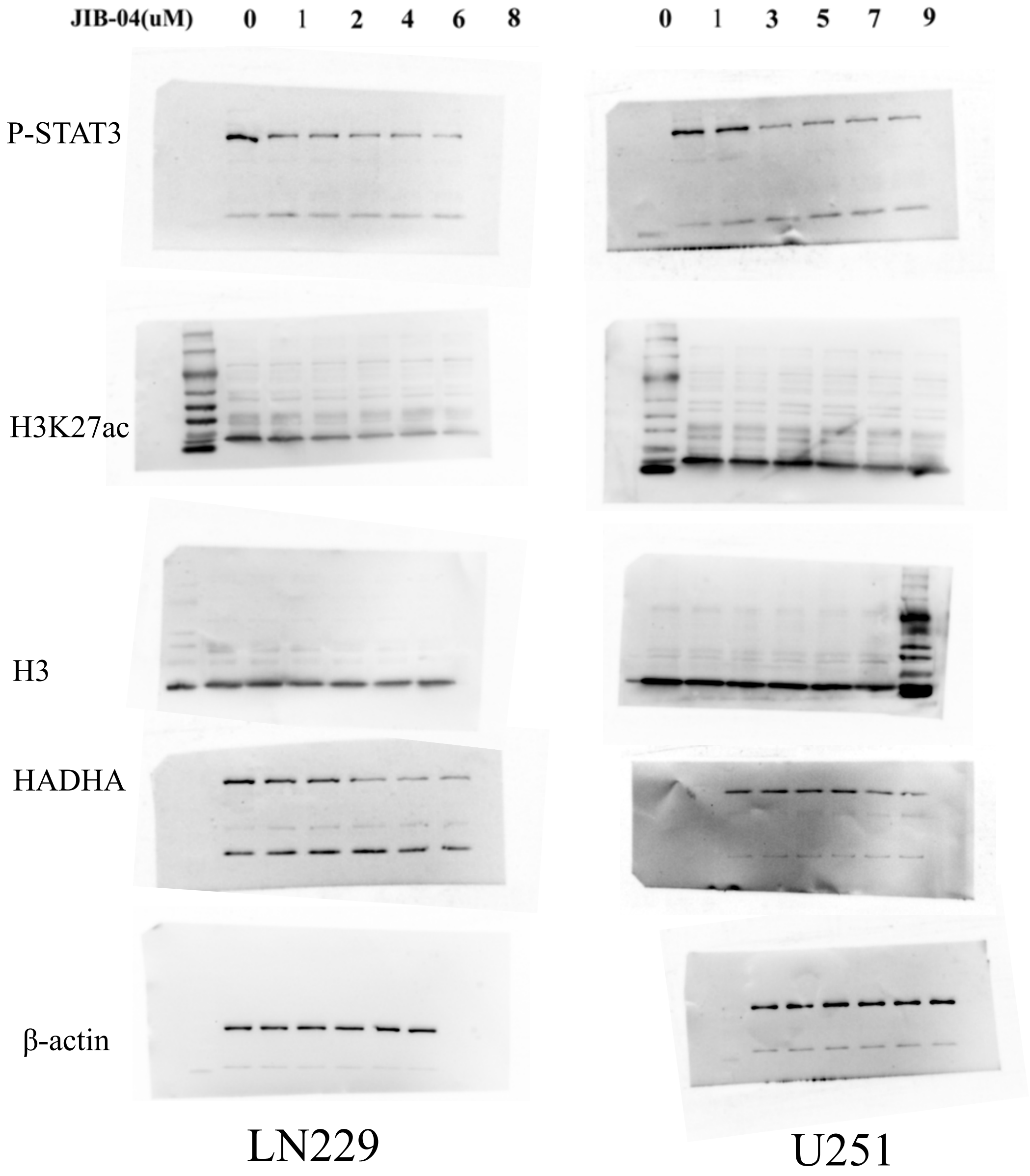

JIB-04(uM)
0
1
2
4
6
8
0
1
3
5
7
9
P-STAT3
H3K27ac
H3
HADHA
β-actin
LN229
U251
