# Supplementary material for: HADHA-mediated regulation of JAK/STAT3 signaling in glioblastoma: a metabolic-epigenetic axis
Source: Cell Death Discov. 2025 Aug 1;11:361. doi: 10.1038/s41420-025-02660-0 (PMC12316893; doi:10.1038/s41420-025-02660-0)
Supplement: Supplementary file 4 — Supplementary Table 1 [file 41420_2025_2660_MOESM4_ESM.docx]

**Supplementary Table 1**. Primers and probes used for qRT-PCR

| Gene | Sequences |  |
| --- | --- | --- |
| HADHA  ACTIN | F: 5′- ACCCAGGCATGTGGAATATG -3′  R: 5′- GTTGCTCCCAGCAGGATTAT -3′  F: 5′- CCTCCATCCTGGCCTCGCTGT -3′  R: 5′- GCTGTCACCTTCACCGTTCC -3′ | |
