# Supplementary material for: HADHA-mediated regulation of JAK/STAT3 signaling in glioblastoma: a metabolic-epigenetic axis
Source: Cell Death Discov. 2025 Aug 1;11:361. doi: 10.1038/s41420-025-02660-0 (PMC12316893; doi:10.1038/s41420-025-02660-0)
Supplement: Supplementary file 5 — Supplementary Table 2 [file 41420_2025_2660_MOESM5_ESM.docx]

**Supplementary Table 2 Specific information about the primary antibody**

| **Primary antibody** | **company** | **molecular weight** | **Article no.** | **Dilutions（WB）** | |  |
| --- | --- | --- | --- | --- | --- | --- |
| HADHA  JAK  P-JAK  STAT3  P-STAT3  H3K27ac  H3  β-Actin | HUABIO  CST  CST  Santa cruz  Santa cruz  Epizyme  Abways  Servicebio | 79 kDa  125 kDa  125 kDa  88 kDa  88 kDa  17 kDa  17 kDa  42 kDa | HA721652  3230  3771  sc-293151  sc-81523  R012098  CY6587  GB15003 | 1:1000  1:1000  1:500  1:1000  1:1000  1:1000  1:2000  1:5000 |  | |
