# Supplementary material for: HADHA-mediated regulation of JAK/STAT3 signaling in glioblastoma: a metabolic-epigenetic axis
Source: Cell Death Discov. 2025 Aug 1;11:361. doi: 10.1038/s41420-025-02660-0 (PMC12316893; doi:10.1038/s41420-025-02660-0)
Supplement: Supplementary file 6 — Supplementary Table 3 [file 41420_2025_2660_MOESM6_ESM.docx]

**Supplementary Table 3**. Primers used for Chip-qPCR

| Gene | Sequences |  |
| --- | --- | --- |
| Primer 1  Primer 2  Primer 3  Primer 4 | F: 5′- ACTTCCTCCGCTTGCC -3′  R: 5′- AGCCTACCCACGACCAG -3′  F: 5′- CGGCAGGAGTGAGGGACAGT -3′  R: 5′- AGCGGGAACAGGGCAAG -3′  F: 5′- GTGGTGGCGAGGTTGAGA -3′  F: 5′- TGCTCACGCAGAAACTGA -3′  F: 5′- GTGACTCCGCCTGCTTTG -3′  F: 5′- TCTTCTGCATTCGCCTGT -3′ | |
